# Searching for new therapeutic options for the uncommon pathogen *Mycobacterium chimaera*: an open drug discovery approach

**DOI:** 10.1016/S2666-5247(21)00326-8

**Published:** 2022-05

**Authors:** Daire Cantillon, Aaron Goff, Stuart Taylor, Emad Salehi, Katy Fidler, Simon Stoneham, Simon J Waddell

**Affiliations:** a Department of Global Health and Infection, Brighton and Sussex Medical School, University of Sussex, Falmer, UK; b Clinical and Experimental Medicine, Brighton and Sussex Medical School, University of Sussex, Falmer, UK; c School of Pharmacy, University of Sussex, Falmer, UK; d Department of Microbiology and Infection, Royal Sussex County Hospital, Brighton, UK; e Academic Department of Paediatrics, Royal Alexandra Children's Hospital, Brighton, UK

## Abstract

**Background:**

*Mycobacterium chimaera* is a slowly growing non-tuberculous mycobacterium associated with outbreaks of fatal infections in patients after cardiac surgery, and it is increasingly being detected in patients with chronic lung conditions. *M chimaera* can cause disseminated disease, osteomyelitis, and chronic skin or soft-tissue infections. We aimed to find new inhibitory compounds and drug repurposing opportunities for *M chimaera*, as current therapeutic options often result in poor outcomes.

**Methods:**

In an open drug discovery approach, we screened the Medicines for Malaria Venture (MMV) Pathogen Box to assess the in-vitro antimicrobial drug susceptibility of *M chimaera* compared with the antimicrobial drug susceptibility of the slowly growing, major human pathogen *Mycobacterium tuberculosis*, and the rapidly growing *Mycobacterium abscessus* reference strains. Compounds identified from an initial resazurin microtitre cell viability assay screen were further characterised by determining the minimum inhibitory concentration (MIC) of MMV Pathogen Box compounds against *M chimaera*; and the MICs of a panel of 20 drugs commonly used to treat mycobacterial infections against *M tuberculosis, M abscessus*, and *M chimaera*. We also assessed the time-kill kinetics of doxycycline, clarithromycin, ethambutol, and rifabutin against *M chimaera*.

**Findings:**

*M chimaera* was inhibited by 21 (5%) of 400 compounds in the Pathogen Box. Ten compounds were active against all three mycobacteria. MMV675968, with activity against slowly growing mycobacteria that probably targets folate metabolism, had a mean MIC of 2·22 μM (0·80 μg/mL) against *M chimaera*. Antimicrobial susceptibility testing showed that oxazolidinones such as linezolid (mean MIC 3·13 μg/mL) were active against *M chimaera* and that bedaquiline was the most potent compound (mean MIC 0·02 μg/mL). Doxycycline, a broad-spectrum antimicrobial drug with excellent tissue penetration properties, also inhibited *M chimaera* with a mean MIC of 6·25 μg/mL.

**Interpretation:**

Molecular diagnostics present an opportunity for more effective, targeted drug therapies—treating bacterial infections at the species level. Using an open drug discovery platform, we identified compounds that inhibit the newly recognised pathogen *M chimaera*. The existing evidence base is poor and the option for expensive drug discovery is improbable; therefore, we have also found options for drug repurposing. Future in-vivo efficacy studies will reveal whether these findings result in new, targeted treatment regimens for *M chimaera*.

**Funding:**

Wellcome Trust, National Centre for the Replacement, Refinement and Reduction of Animals in Research (NC3Rs), and the University of Sussex Junior Research Associate scheme.

## Introduction

*Mycobacterium intracellulare* subspecies *chimaera* (*M chimaera*) is a slowly growing non-tuberculous mycobacterium of the *Mycobacterium avium* complex. It was first recognised in 2004, and interest in it developed quickly after numerous outbreaks of prosthetic valve endocarditis and disseminated disease in patients who had undergone cardiopulmonary bypass surgery.^1^ Whole-genome sequencing of isolates suggested a single common source, which was traced to heater–cooler units used during cardiac surgery.^2^ *M chimaera* disease onset is typically more than 1 year after surgery, it is resistant to antimicrobial drug therapy, and it results in high mortality.^1^ Cardiac disease is the greatest cause of death globally and it is rising rapidly in low-income and middle-income countries; as the incidence of cardiac surgery increases, so too will the risk of nosocomial infection.

*M chimaera* disease after cardiac surgery can present with endocarditis, chronic sternal wound infection, or disseminated infection.^3^ In addition, *M chimaera* is increasingly being detected in patients with chronic lung disease, such as cystic fibrosis or chronic obstructive pulmonary disease, and causes disseminated disease, osteomyelitis, and chronic skin or soft-tissue infections.^4^ The number of *M chimaera* cases is probably under-reported, and the misdiagnoses of infections result in delayed and inappropriate treatment.^5^, ^6^ Improved molecular diagnostics will result in more frequent identification of uncommon mycobacterial infections, which offers opportunities to treat patients using specialised, effective drug regimens tailored to each bacterium.

**Research in context**

**Evidence before this study**

Non-tuberculous mycobacterial infections are hard to treat, requiring long-term multidrug regimens that often result in poor patient outcomes. *Mycobacterium chimaera* is a newly identified, slowly growing, non-tuberculous mycobacterium responsible for prosthetic valve endocarditis, opportunistic pulmonary infections, neurological, and disseminated disease. We searched PubMed for peer-reviewed studies published from database inception to July 1, 2021, with no language restrictions, using the terms “*Mycobacterium chimaera”*; “*Mycobacterium avium* complex”; “nontuberculous mycobacterium”; “mycobacterium drug discovery”; and “nontuberculous mycobacteria treatment”. Reference lists from identified studies were also screened for peer-reviewed articles of interest. *M chimaera* is listed under the treatment guidelines for *Mycobacterium avium* complex. Azithromycin, rifampicin, and ethambutol, with the addition of amikacin dependent on disease severity, is recommended for *M avium* complex treatment for a minimum of 12 months after conversion to culture negative. There are very few laboratory or clinical drug efficacy studies focused specifically on *M chimaera*, and there is little opportunity to evaluate new drug combinations in clinical trials. The Pathogen Box compounds have been tested against *Mycobacterium tuberculosis* and *M avium* but not *M chimaera*. Doxycycline has been shown to have activity against *M tuberculosis*, and it is an option for treating *Mycobacterium abscessus* pulmonary disease. There has been minimal exploration of its use in *M avium* complex. Bedaquiline has been suggested as a new drug option for non-tuberculous mycobacteria, but definitive clinical evidence of efficacy is missing. Linezolid is not recommended for *M avium* complex, although new generation oxazolidinones are under investigation.

**Added value of this study**

This study identified several antimicrobial drug repurposing options for *M chimaera*, reference strain NCTC13781, including oxazolidinones, bedaquiline, and doxycycline—a lipophilic drug with excellent tissue penetration properties. These findings are based on in-vitro antimicrobial drug susceptibility testing against reference strains of rapidly growing and slowly growing mycobacteria. The study also discovered Pathogen Box compounds with druggable physiochemical properties that inhibited *M chimaera* and *M abscessus*. These data are freely available in an open research approach to drug discovery.

**Implications of all the available evidence**

It remains a challenge to make evidence-based treatment decisions for rare bacterial diseases. Increased molecular definition of bacterial infections increases the complexity of this task; however, it also offers opportunities to improve patient outcomes by improving targeted antimicrobial therapy. Repurposing antimicrobial drugs with known pharmacokinetic and favourable safety profiles remains the most feasible pathway to improve treatment for non-tuberculous mycobacterial diseases. This study suggests new avenues for open-source drug discovery and drug repurposing for *M chimaera*. Prospective clinical trials and efficacy studies will be required to validate these findings before they influence clinical practice of this uncommon but often fatal mycobacterial infection.

The American Thoracic Society, European Respiratory Society, European Society of Clinical Microbiology and Infectious Diseases, and Infectious Diseases Society of America joint clinical practice guideline recommends the treatment of *M avium* complex pulmonary disease on the basis of disease severity, and it does not distinguish treatment by subgroup speciation.^7^ In severe disease, treatment involves daily rifampicin, ethambutol, azithromycin, and occasionally addition of intravenous or nebulised amikacin. Therapy should be continued for up to one year after conversion to culture negative. In one UK study, 15 (60%) of 25 patients with postoperative *M chimaera* died; ten of those who died received treatment for *M chimaera*.^8^ The high mortality rate despite 12 months of antimicrobial therapy highlights the challenge of treating *M chimaera* in at-risk patients with multiple comorbidities.

Antimicrobial drug susceptibility testing of subspeciated *M avium* complex clinical isolates revealed broadly similar drug susceptibilities, with some minor differences between isolates.^9^ Schulthess and colleagues also reported comparable drug susceptibilities between *M chimaera* and *M avium*.^10^ However, the microbiological and clinical diversity of *M avium* complex infections suggests that effective targeted therapeutic options might exist.

Using an open drug discovery approach, this study aimed to address the challenge of delivering new treatment options for *M chimaera*, in which the existing evidence base is poor and the rational for expansive drug discovery research is unjustified.

## Methods

### Study design and cultures

We adopted an open drug discovery approach for this study, which was done between Aug, 8, 2018, and March, 1, 2020, in the Department of Global Health and Infection, Brighton and Sussex Medical School, University of Sussex, UK.

*M chimaera* reference strain NCTC13781, *Mycobacterium abscessus* reference strain ATCC19977, and *Mycobacterium tuberculosis* reference strain H37Rv (from the National Collection of Type Cultures, Public Health England) were cultured in Middlebrook 7H9 broth (Sigma Aldrich, St Louis, MO, USA) supplemented with albumin dextrose catalase (ADC; 10% volume per volume [v/v]) and Tween 80 (0·05% v/v; Sigma Aldrich, St Louis, MO, USA) at 37°C. Optical density was measured using a spectrophotometer at absorbance 600 nm. Colony forming units (CFUs) were determined by serially diluting cultures onto Middlebrook 7H10 agar (Sigma Aldrich, St Louis, MO, USA) supplemented with 0·5% glycerol and oleic acid albumin dextrose catalase (OADC; 10% v/v) and incubated at 37°C for 4 weeks for *M tuberculosis,* 3 weeks for *M chimaera,* and 3 days for *M abscessus*. Laboratory work with *M chimaera* and *M abscessus* were conducted in biosafety level-2 laboratories; work with *M tuberculosis* was conducted in a biosafety level-3 laboratory. This study did not require ethics permissions or Institutional Review Board approval.

### Procedures

#### Assay validation for M chimaera

We validated the resazurin microtitre cell viability assay (REMA) as a screening method, REMA (CellTiter-Blue, Promega, Madison, WI, USA) was used to determine the antimicrobial activities of the drugs.^11^ Log phase *M chimaera* cultures were diluted to 1 × 10^5^–5 × 10^5^ CFU/mL, inoculated into a series of drug-free wells containing 2% v/v dimethyl sulfoxide (Sigma Aldrich, St Louis, MO, USA) and into wells containing 2·5 μg/mL rifampicin (50 times the minimum inhibitory concentration [MIC]; Sigma Aldrich, St Louis, MO, USA), and incubated at 37°C for 7 days. A 2% dimethyl sulfoxide concentration was selected to match the final dimethyl sulfoxide concentration of the Pathogen Box screen. To determine antimicrobial activity, CellTiter-Blue was added at a final concentration of 10% v/v and incubated for 16 h. Fluorescence was measured at excitation 580–640 nm and emission 520 nm, using a Glomax Discover plate reader (Promega, Madison, WI, USA). Fluorescence data were corrected for background using media-dimethyl sulfoxide bacteria-free controls. Experiments were repeated a minimum of three times and the datapoints were pooled.

#### Pathogen Box whole cell screen for antimycobacterial activity

We screened the Medicines for Malaria Venture (MMV) Pathogen Box to assess the in-vitro antimicrobial drug susceptibility of *M chimaera* compared with the antimicrobial drug susceptibility of slowly growing, major human pathogen *M tuberculosis*, and the rapidly growing *M abscessus*. The Pathogen Box contained 400 drug-like compounds active against various neglected diseases, including 21 reference compounds with known antimicrobial activity; the concentration of the compounds provided was 10 mM in 100% dimethyl sulfoxide.^12^ Log phase cultures of *M chimaera, M abscessus*, and *M tuberculosis* were diluted to 1 × 10^5^–5 × 10^5^ CFU/mL and added to 96-well plates containing Pathogen Box compounds at a final concentration of 20 μM (2% v/v dimethyl sulfoxide). Drug-free controls were inoculated alongside drug controls of 5 μg/mL rifampicin (>1000 times the MIC against *M tuberculosis;* 100 times the MIC against *M chimaera*) or 3·9 μg/mL MIC clarithromycin (3 times the MIC against *M abscessus*). Plates were incubated at 37°C for 3 days for *M abscessus* and 7 days for *M chimaera* and *M tuberculosis*. Antimicrobial activity was assessed using REMA. Background fluorescence was corrected by adjusting measurements to media-dimethyl sulfoxide bacteria-free controls. Hits were classed as compounds that inhibited bacterial growth by 70% or more compared with drug-free controls (appendix p 2).

#### Determination of MICs

We measured the MICs of MMV compounds that had hits against *M chimaera*, and to repurpose commonly used antimycobacterial drugs for *M chimaera* we measured the MIC of 20 drugs used in first-line or second-line tuberculosis treatment, or that treat non-tuberculous mycobacteria. All drugs were prepared as 10 mg/mL stock solutions in sterile dimethyl sulfoxide, except for rifampicin and rifabutin, which were prepared with 90% weight per volume methanol, and aminoglycosides, which were prepared in sterile water. Single use drug aliquots were stored at –20°C. MICs were determined using a microbroth dilution method as recommended by the Clinical and Laboratory Standards Institute (CLSI) for non-tuberculous mycobacteria,^13^ but with modifications (appendix p 3). Middlebrook 7H9 broth (0·05% Tween 80, 10% ADC) was used as the culture media to enable comparison between the three mycobacteria. Two-fold dilutions of the 20 antimicrobial compounds or three-fold dilutions of the MMV compounds were prepared in 96-well microtitre plates and inoculated with mycobacteria to a final concentration of 1 × 10^5^–5 × 10^5^ CFU/mL. The plates were incubated at 37°C for 3 days for *M abscessus* and 7 days for *M chimaera* and *M tuberculosis.*

MIC values were estimated by REMA after 16 h incubation with CellTiter-Blue. Fluorescence data were corrected for background using media-only controls. The MIC was defined as the minimum concentration of compound required to inhibit bacterial growth by 90% or more, except for ethambutol and ethionamide for which the MIC was calculated as the lowest concentration of the drug to consistently inhibit growth because 90% inhibition could not be obtained. Positive controls were drug-free wells that were included to assay uninhibited mycobacterial growth. Negative controls were bacteria-free, drug-free wells that were included to define background fluorescence and assess sterility of the assay. Experiments were repeated a minimum of three times, with triplicate datapoints per experiment. We calculated the mean MIC from a minumum of nine datapoints.

#### M chimaera time-kill assay

Time-kill assays of doxycycline, clarithromycin, ethambutol, and rifabutin were conducted for *M chimaera.* Log phase Middlebrook 7H9 broth cultures, corresponding to 10^8^ CFU/mL in 20 mL volumes, were treated with two times or ten times the drug MIC or dimethyl sulfoxide carrier control and incubated at 37°C with shaking at 180 revolutions per min. Optical density and CFU counts were determined at 0, 4, 7, 10, and 14 days.

### Statistical analysis

REMA was validated as a screening method by calculating the estimated Z factor, where the degree of separation between positive and negative controls determines the reliability of the assay.^14^ A value between 0·5 and 1·0 indicates that an assay is statistically reliable enough to discriminate between positive and negative control wells; the closer to 1·0, the more robust the assay. The estimated Z factor for the Pathogen Box REMA screen was determined using the formula:

$$Zfactor=1-\frac{3(\sigma \text{p}+\sigma \text{n})}{\left|\mu \text{p}-\mu \text{n}\right|}$$

In this formula, σ*p* is the standard deviation of the positive controls; σ*n* is the standard deviation of the negative controls; *μp* and *μn* are the means of the positive and negative controls, respectively. The *M chimaera* REMA assay estimated Z factor comparing drug-free bacilli with rifampicin-treated bacilli was 0·87, demonstrating that this screening method was statistically robust and appropriate for assessing antimicrobial drug activity. Error bars were assigned to MIC plots by applying SD of pooled technical replicates from three independent experiments in GraphPad Prism (version 9).

### Role of the funding source

The funders of the study had no role in study design, data collection, data analysis, data interpretation, or writing of the report.

## Results

**Figure 1:**
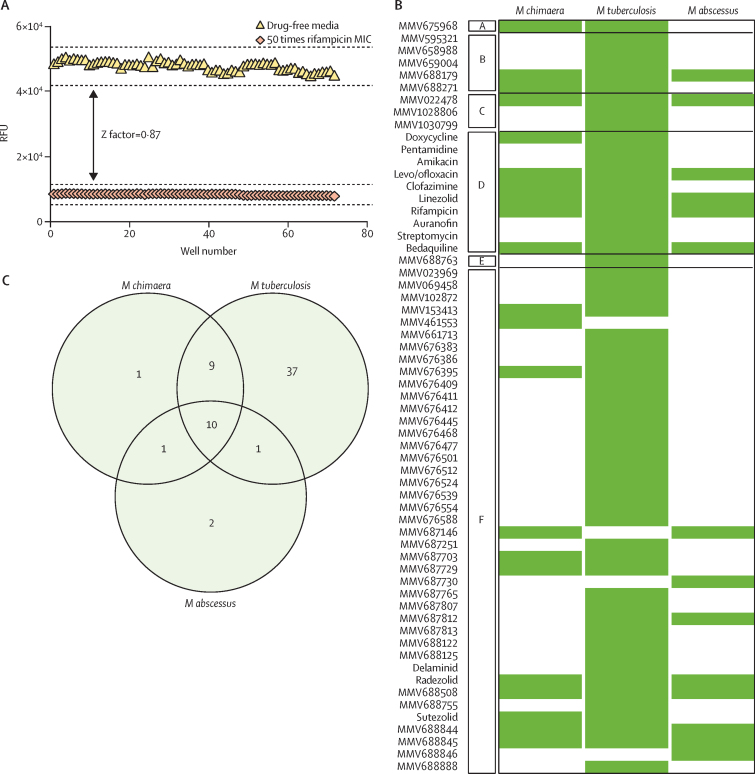
Antimicrobial compound hits identified from screening the Pathogen Box against *M chimaera* A) Validation of the REMA screening approach for *M chimaera*, demonstrating an estimated Z factor of 0·87. 50 times rifampicin mean MIC (2·5 μg/mL; red diamond) was compared to 2% dimethyl sulfoxide drug-free media (yellow triangle) in the REMA assay. Data points from three independent experiments were pooled. (B) The 400 Pathogen Box compounds screened against *M chimaera, M tuberculosis*, and *M abscessus* at 20 μM with cell survival quantified by REMA. Any compound that inhibited growth by 70% or more were classed as a hit (green bar). Compounds have been classified by MMV as A for cryptosporidiosis; B for kinetoplastids; C for malaria; D for reference compounds; E for schistosomiasis; and F for tuberculosis. (C) Venn diagram describing the overlap in antimicrobial compound hits against *M chimaera, M abscessus*, and *M tuberculosis*. The compounds are detailed as a drug name or by the MMV identifier. *M chimaera*=*Mycobacterium chimaera*. *M tuberculosis*=*Mycobacterium tuberculosis*. *M abscessus*=*Mycobacterium abscessus*. MMV=Medicines for Malaria Venture. MIC=minimum inhibitory concentration. RFU=relative fluorescence units. REMA=resazurin microtitre cell viability assay.

**Figure 2:**
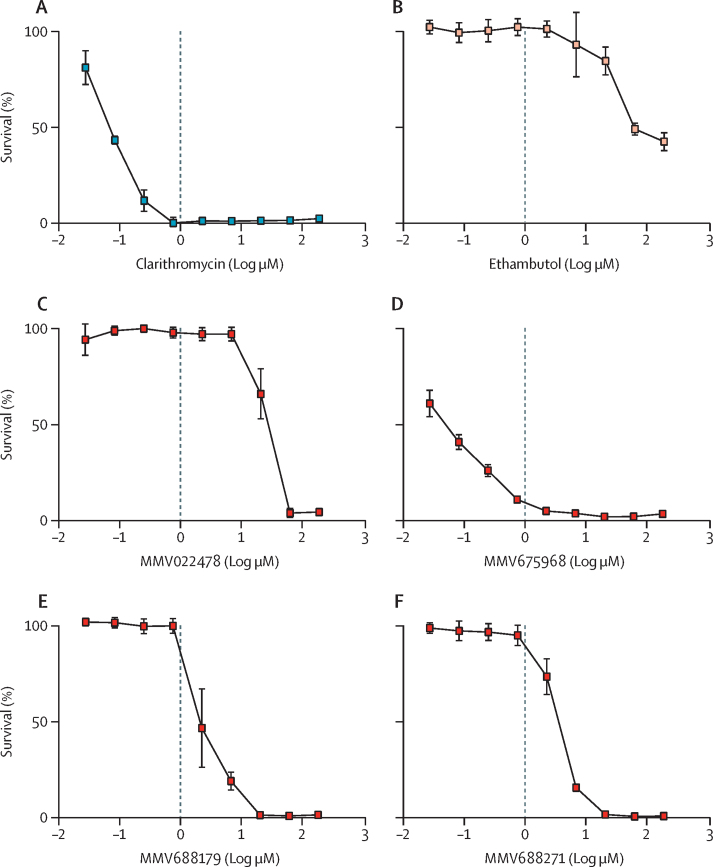
Dose responsive inhibition of *M chimaera* by selected Pathogen Box compounds MICs were determined against log phase *M chimaera*. *M chimaera* was exposed to a dilution series of each MMV compound, alongside clarithromycin and ethambutol as comparators, from 0·03 to 180 μM. Percentage survival relative to drug-free controls are plotted, where drug-free controls (not plotted) equate to 100% survival. Each data point is the mean of three biological replicates, with error bars as SD. MMV=Medicines for Malaria Venture. *M chimaera*=*Mycobacterium chimaera*. MIC=minimum inhibitory concentration.

Of the 400 Pathogen Box compounds, 21 compounds showed activity in the REMA screen against *M chimaera*, 57 compounds were active against *M tuberculosis*, and 14 compounds were active against *M abscessus* (figure 1; appendix pp 7–16). Only ten compounds in the Pathogen Box were active against all three mycobacteria. Of the *M tuberculosis* hits, 11 (19%) of 57 also showed activity against *M abscessus,* whereas 19 (33%) of 57 showed activity against *M chimaera*. 19 (90%) of 21 compounds that inhibited *M chimaera* were also active against *M tuberculosis*. 15 of 21 initial screening hits against *M chimaera* were drug-like compounds featured in the MMV Pathogen Box; the other six were Pathogen Box reference compounds (ie, established antimicrobial drugs). These were rifampicin, part of the first-line treatment for drug sensitive tuberculosis and *M avium* complex; clofazimine and linezolid used in non-tuberculous mycobacterial pulmonary disease; bedaquiline and levo/ofloxacin options for the treatment of multidrug-resistant tuberculosis; and doxycycline, a broad-spectrum tetracycline antimicrobial drug that was a potent inhibitor of *M chimaera* inhibiting growth by approximately 90%. 11 (73%) of 15 MMV hits were previously defined as active against mycobacteria in a screen of *M tuberculosis*: MMV688327, MMV688508, MMV676395, MMV688845, MMV688756, MMV687703, MMV688844, MMV687146, MMV461553, MMV153413, and MMV687729.^15^ The four remaining compounds active against *M chimaera* were not classed as anti-tuberculosis drugs and further compound was requested from MMV to determine MICs (MMV675968, MMV022478, MMV688179, and MMV688271); two were characterised as anti-kinetoplastid, one as anti-malarial, and one as anti-cryptosporidiosis. MICs confirmed dose–responsive activity of these compounds against *M chimaera* (figure 2). The mean MICs were 2·22 μM (0·80 μg/mL) for MMV675968, 20 μM (9·52 μg/mL) for isomers MMV688179 and MMV688271, and 60 μM (32·76 μg/mL) for MMV022478. All four compounds display favourable druggable characteristics (appendix pp 17–18), thus contributing to the portfolio of antimicrobial activities for these compounds.

Since the primary screen revealed that the majority of *M chimaera* hits were also active against *M tuberculosis,* we measured MICs in *M chimaera, M abscessus*, and *M tuberculosis* for 20 first-line and second-line anti-tuberculosis drugs alongside drugs that have been prescribed to treat non-tuberculous mycobacteria (table). Isoniazid showed no activity against *M chimaera* or *M abscessus* (mean MICs >200 μg/mL), despite its use clinically for clarithromycin-resistant *M chimaera* infections.^16^ Ethambutol, a first-line agent for *M chimaera*, showed activity against *M chimaera* with a mean MIC of 6·25 μg/mL. However, this drug only inhibited growth by 70–80%, even at the highest concentration tested of 200 μg/mL. A similar result was observed for ethionamide, with *M chimaera* growth inhibited by 70–80% at concentrations of 25 μg/mL. Neither of these cell wall-targeting agents inhibited *M abscessus* (mean MICs >200 μg/mL). Rifampicin and rifabutin showed potent activity against *M chimaera* (mean MIC 0·05 μg/mL for both), with rifabutin more active than rifampicin in *M abscessus.* Bedaquiline was the most potent antimicrobial drug tested against *M chimaera* with a mean MIC of 0·02 μg/mL; it was also active against *M abscessus* with a mean MIC of 0·39 μg/mL. Linezolid, a component of regimens used to treat pulmonary *M avium* complex disease in cases for which resistance to first-line agents is identified, was effective against all three mycobacteria, with mean MICs of 3·13 μg/mL against *M chimaera*, 6·25 μg/mL against *M abscessus*, and 1·56 μg/mL against *M tuberculosis*. The next generation oxazolidinones, radezolid and sutezolid, also inhibited *M chimaera*. Clofazimine and aminosalicylic acid showed activity against *M chimaera* with a mean MIC of 0·20 μg/mL and 6·25 μg/mL, respectively.

**Table:** MICs of commonly prescribed antimycobacterial drugs against *Myobacterium chimaera, Mycobacterium tuberculosis*, and Mycobacterium abscessus

	**Mean MIC against *M chimaera*, mu;g/mL**	**Mean MIC against *M tuberculosis*, mu;g/mL**	**Mean MIC against *M abscessus*, mu;g/mL**
**Aminoglycoside**			
Amikacin	5·210 (1·800)	0·195 (0)*	10·417 (3·610)
Streptomycin	3·130 (0)	0·156 (0)*	12·500 (0)
Tobramycin	0·780 (0)	0·200 (0)*	10·417 (3·610)
**Aminosalicylate**			
Aminosalicylic acid	6·250 (0)	0·390 (0)*	>200 (0)
**Carbapenem**			
Imipenem	8·333 (3·610)	1·560 (0)*	10·417 (3·610)
**Diarylquinoline**			
Bedaquiline	0·020 (0)*	0·049 (0)	0·390 (0)
**Ethylenediamine**			
Ethambutol	6·250 (0)	3·130 (0)*	>200 (0)
**Fluoroquinolone**			
Ciprofloxacin	0·780 (0)	0·313 (0)*	1·560 (0)
Moxifloxacin	0·200 (0)*	0·780 (0)	1·040 (0·45)
Ofloxacin	2·607 (0·910)	0·625 (0)*	0·625 (0)*
**Hydrazide**			
Isoniazid	>200 (0)	0·390 (0)*	>200 (0)
**Macrolide**			
Azithromycin	10·417 (3·610)*	83·333 (28·870)	25·000 (0)
Clarithromycin	0·390 (0)*	1·300 (0·450)	1·040 (0·450)
Erythromycin	8·333 (3·610)*	16·667 (5·770)	16·667 (7·220)
**Oxazolidinone**			
Linezolid	3·130 (0)	1·560 (0)*	6·250 (0)
**Phenazine**			
Clofazimine	0·200 (0)*	1·300 (0·45)*	6·250 (0)
**Rifamycin**			
Rifampicin	0·050 (0)	0·003 (0)*	10·417 (3·61)
Rifabutin	0·050 (0)	0·003 (0)*	1·560 (0)
**Tetracycline**			
Doxycycline	6·250 (0)	3·125 (0)*	50·000 (0)
**Thioamide**			
Ethionamide	25·000 (0)	6·250 (0)*	>200 (0)

Data are mean (SD) unless indicated otherwise. There was no more than one dilution difference in MICs between biological replicates. MICs were determined using the microbroth dilution method from three independent biological replicates. The table is ordered by drug class. MIC=minimum inhibitory concentration.

* The lowest MIC value for each drug.

Aminoglycosides are sometimes added to *M chimaera* drug regimens, and they are important second-line drugs for multidrug resistant tuberculosis. For *M tuberculosis*, the amikacin mean MIC was 0·195 μg/mL and the streptomycin mean MIC was 0·156 μg/mL. However, the concentrations required for non-tuberculous mycobacteria were higher with 27 times more amikacin (5·21 μg/mL) and 20 times more streptomycin (3·13 μg/mL) required for *M chimaera*, and 53 times more amikacin (10·42 μg/mL) and 80 times more streptomycin (12·5 μg/mL) required for *M abscessus.* Amikacin and tobramycin can be delivered as nebulised drugs, and the tobramycin mean MIC for *M chimaera* (0·78 μg/mL) was almost seven times lower than the mean MIC of amikacin (5·21 μg/mL). The fluoroquinolones ciprofloxacin, moxifloxacin, and ofloxacin were all active against *M chimaera*, with a ciprofloxacin mean MIC of 0·78, a moxifloxacin mean MIC of 0·20, and an ofloxacin mean MIC of 2·61 μg/mL. The macrolide clarithromycin is a first-line drug for *M chimaera*; and clarithromycin was the most potent macrolide with a mean MIC of 0·39 μg/mL, azithromycin had a mean MIC of 10·42 μg/mL, and erythromycin had a mean MIC of 8·33 μg/mL. Clarithromycin was also the most active macrolide against *M tuberculosis* (1·30 μg/mL mean MIC) and *M abscessus* (1·04 μg/mL mean MIC). The order of macrolide in-vitro efficacy was clarithromycin followed by erythromycin, and then azithromycin for all three mycobacteria. Imipenem, a carbapenem that is highly stable against many β lactamases, and has been used to treat *M chimaera*, had a mean MIC of 8·33 μg/mL.

**Figure 3:**
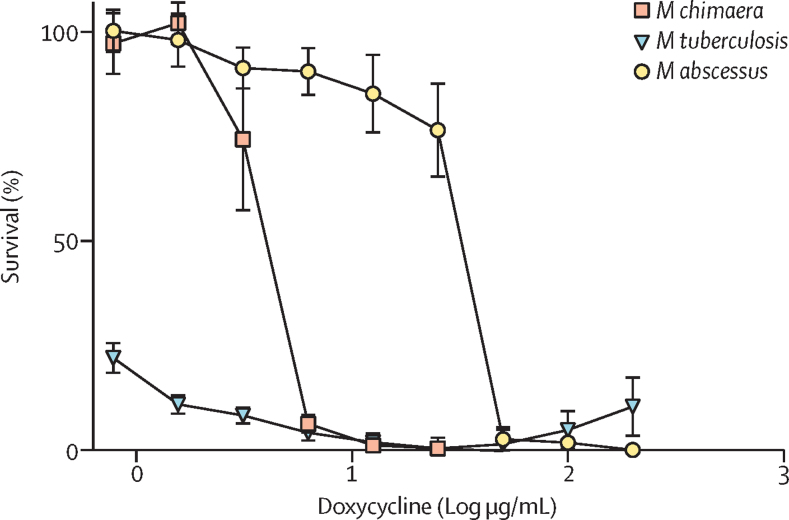
Doxycycline is active against *M chimaera* *M chimaera*=*Mycobacterium chimaera. M tuberculosis*=*Mycobacterium tuberculosis*. *M abscessus*=*Mycobacterium abscessus.* MIC=minimum inhibitory concentration. Log phase *M chimaera* (red square), *M tuberculosis* (blue triangle), and *M abscessus* (yellow circle) were exposed to doxycycline over ten two-fold dilutions (from 0·78–200 μg/mL) to determine the MICs. The MICs were 6·25 μg/mL against *M chimaera*, 3·125 μg/mL against *M tuberculosis* and 50 μg/mL against *M abscessus*. Percentage survival relative to drug-free controls are plotted; drug-free controls (not plotted) equate to 100% survival. Data points are the mean of three biological replicates and SD.

**Figure 4:**
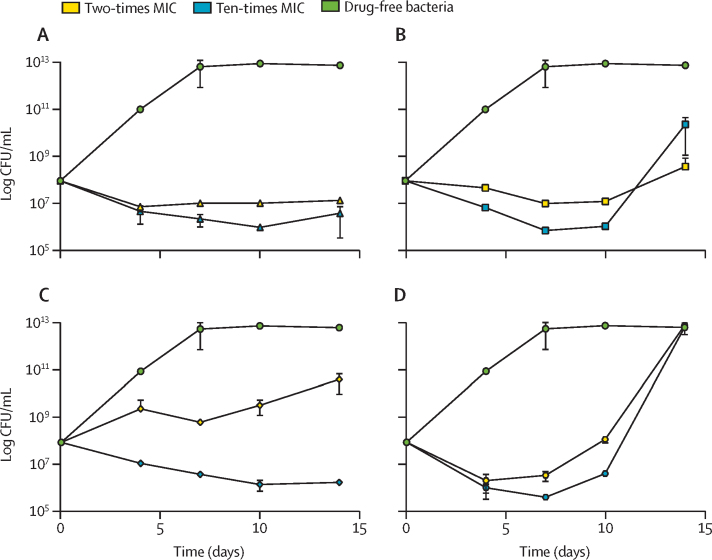
Time-kill kinetics of doxycycline, clarithromycin, ethambutol, and rifabutin against *Mycobacterium chimaera* (A) Exponential phase *Mycobacterium chimaera* was exposed to two-times doxycycline MIC (yellow triangle) or ten-times doxycycline MIC (blue triangle). (B) Two-times clarithromycin MIC (yellow square) and ten-times clarithromycin MIC (blue square). (C) Two-times ethambutol MIC (yellow diamond) and ten-times ethambutol MIC (blue diamond). (D) Two-times rifabutin MIC (yellow hexagon) or ten-times rifabutin MIC (blue hexagon) compared with drug-free bacteria (green circle). CFU/mL were determined over 14 days. Each data point is the mean of three biological replicates with error bars as SD. MIC=minimum inhibitory concentration. CFU=colony forming unit.

Doxycycline, which has been used to treat *M abscessus*, was identified as a hit in our Pathogen Box screen. We determined a doxycycline mean MIC against *M chimaera* of 6·250 μg/mL*,* 3·125 μg/mL against *M tuberculosis*, and 50 μg/mL against *M abscessus* (figure 3). The slowly growing *M chimaera* and *M tuberculosis* were more sensitive to doxycycline, whereas the rapidly growing *M abscessus* was only inhibited by doxycycline at covncentrations of 50 μg/mL or higher. Doxycycline has been widely reported to be bacteriostatic and we verified this by measuring time-kill kinetics alongside clarithromycin, ethambutol, and rifabutin (the first-line drugs for *M chimaera*). Doxycycline was bacteriostatic against *M chimaera* (figure 4A), and there was no regrowth of bacteria over time when treated with two times the doxycycline mean MIC (12·50 μg/mL) or ten times the doxycycline mean MIC (62·50 μg/mL). *M chimaera* regrowth after 14 days was observed when treated with two times the clarithromycin mean MIC (0·78 μg/mL), ten times the clarithromycin mean MIC (3·90 μg/mL), two times the rifabutin mean MIC (0·10 μg/mL), ten times the rifabutin mean MIC (0·50 μg/mL), and two times the ethambutol mean MIC (12·50 μg/mL; figure 4B–D).

## Discussion

The development of molecular diagnostic tools that differentiate bacterial infections at the species level offers unprecedented opportunities to personalise more effective antimicrobial drug therapies to treat rare pathogens. To realise effective therapies, simple discovery pathways must signpost potential therapeutic combinations. We optimised a viability assay for *M chimaera* and screened the open drug discovery MMV Pathogen Box,^12^ comprising 400 drug-like compounds with antimicrobial activity, low cytotoxicity, and favourable drug kinetics against reference strains of three pathogenic mycobacteria (*M chimaera, M tuberculosis*, and *M abscessus*). This approach enabled a broad comparison of drug efficacy for *M chimaera* with the rapidly growing *M abscessus* and slowly growing *M tuberculosis*. Our findings also contribute to open-source drug discovery efforts for emerging bacterial threats and neglected tropical diseases. We determined the MICs for Pathogen Box hits alongside established antimicrobial drugs, identifying new antimycobacterial compound activities and drug repurposing options for *M chimaera*.

Of the 21 MMV Pathogen Box compounds that inhibited *M chimaera*, six were reference compounds with established antimicrobial activity, 11 were classed as anti-tuberculosis drugs,^16^ two as anti-kinetoplastid, one as anti-malarial, and one as anti-cryptosporidiosis. We determined the MICs of four hits with novel antimycobacterial activity. MMV675968, a diaminoquinazoline, had a mean MIC of 2·22 μM (0·80 μg/mL) against *M chimaera,* and also inhibited *M tuberculosis*. It is classed as an anti-cryptosporidiosis compound that targets dihydrofolate reductase,^17^ a key enzyme in the mycobacterial cellular processes of folate metabolism and nucleotide synthesis. This enzyme is a target of the tuberculosis drug aminosalicylic acid,^18^ and it is the focus of continued drug discovery efforts. We established an aminosalicylic acid mean MIC of 6·25 μg/mL for *M chimaera*, highlighting the potential of exploiting folate metabolism for *M chimaera* drug development with new and existing chemical entities. MMV022478, with a mean MIC of 60 μM (32·76 μg/mL) against *M chimaera* and activity against *M tuberculosis* and *M abscessus*, is a pyrazolopyrimidine with in-vitro activity against the parasite *Trypanosoma brucei*.^19^ MMV022478 has been shown to inhibit protein kinase C which activates nicotinamide adenine dinucleotide phosphate oxidase in eukaryotic cells, thus inhibiting a range of cellular processes.^20^ MMV688179 and MMV688271 isomers, both with a mean MIC of 20 μM (9·52 μg/mL) against *M chimaera* and possessing activity against *M tuberculosis,* are bisarylguanidiniums that bind to the minor groove of adenine-thymine rich regions of DNA, and they possess activity against a wide range of trypanosomal parasite life stages.^19^ This class of compounds might also have affinity for mammalian DNA,^19^ although they showed low toxicity against human cell lines (appendix p 18).^12^ We identified ten antimicrobial compounds that inhibited all three mycobacterial pathogens screened (MMV688179, MMV022478, MMV688508, MMV68844, MMV68845, and the reference drugs levo/ofloxacin**,** linezolid, rifampicin, bedaquiline, and radezolid). MMV688508, MMV688844, and MMV688845 are already classified as anti-tuberculosis compounds, and MMV688179 and MMV022478 were newly identified to inhibit mycobacteria in this study. Activity against both rapidly growing and slowly growing mycobacteria suggest that these compounds have broad antimycobacterial activity worthy of further investigation.

The MMV Pathogen Box has been applied previously to drug discovery for *M tuberculosis, M abscessus,* and *M avium.*^16^, ^21^ The correlation between drug screening studies for *M abscessus* was high, with 11 of 13 hits from Low and colleagues' study^21^ identified in this study alongside three additional compounds (appendix p 4). However, a comparison of *M avium* with *M chimaera* drug screening studies revealed a lower number of overlapping effective compounds, with 17 of 33 *M avium* hits from Low and colleagues' study found to inhibit *M chimaera* in this study (appendix p 5). The low overlap of hits between *M avium* and *M chimaera* (in comparison to the high similarity between *M abscessus* screens), suggests that *M chimaera* drug susceptibilities might not always match that of *M avium*. Indeed, 16 compounds were identified to have anti-*M avium* activity by Low and colleagues that did not inhibit *M chimaera* in this study.

To repurpose commonly used antimycobacterial drugs for *M chimaera*, we determined the MICs of tuberculosis and non-tuberculous mycobacteria antimicrobial drugs against *M chimaera, M tuberculosis*, and *M abscessus.* Bedaquiline, clofazimine, moxifloxacin, erythromycin, azithromycin, and clarithromycin had the lowest MIC values against *M chimaera* of the three mycobacteria. The oxazolidinones (linezolid, radezolid, and sutezolid), which bind to the ribosome to prevent translation initiation, inhibited *M chimaera*, suggesting that these might be useful drugs*.* The oxazolidinones were also three of the top ten hits against *M avium* in the study by Low and colleagues.^21^ The most effective aminoglycoside against *M chimaera* was tobramycin, which can be administered intravenously or via a nebuliser, which suggests that tobramycin should be compared with amikacin for *M chimaera* pulmonary infections, the currently recommended aminoglycoside. However, unlike amikacin, no tobramycin breakpoints exist for *M avium* complex, and it is not known whether tobramycin MICs correspond to clinical efficacy.

Bedaquiline was the most potent antimicrobial drug tested against *M chimaera* (mean MIC 0·02 μg/mL). Bedaquiline is used safely and effectively to treat multidrug-resistant tuberculosis; its efficacy against slowly growing non-tuberculous mycobacteria has been demonstrated in vitro, with bacteriostatic activity from 0·007 to 0·03 μg/mL and bactericidal activity from 1 to 2 μg/mL.^22^ A bedaquiline-clofazimine combination was shown to be synergistic and additive in vitro against *M chimaera*.^23^ Clinical studies of bedaquiline efficacy against non-tuberculous mycobacteria are limited in number. In a cohort of ten patients with chronic *M avium* complex or *M abscessus* pulmonary disease, treatment with a regimen containing bedaquiline led to a moderate clinical response of 50% of patients reaching conversion to culture negative after six months of treatment.^24^ The cost and potential side-effects in patients with *M chimaera* and multiple comorbidities might also be a concern; however, bedaquiline might prove to be a useful therapeutic option for *M chimaera*.

Doxycycline was demonstrated to have activity against *M chimaera*, with a mean MIC of 6·250 μg/mL. To date, no breakpoints have been set for this drug in *M chimaera*.^9^ Patient case studies have shown that *M chimaera* can spread into bone, limiting treatment options.^1^, ^3^ Doxycycline is highly lipophilic, acting on intracellular bacteria, and it is rapidly distributed in bone and muscle where disseminated *M chimaera* infections can reside. Importantly, doxycycline has an excellent safety profile for long-term use, and it has the pharmacodynamic and pharmacokinetic properties of a potentially effective antimycobacterial agent, including crossing the blood–brain barrier. Doxycycline is recommended for the long-term treatment of several infectious diseases, including Whipple's disease caused by the actinomycete *Tropheryma whipplei*, and it can be added to the continuation phase of *M abscessus* therapy.^7^ Doxycycline was not identified as a hit against *M avium* by Low and colleagues.^21^ This highlights the importance of designing treatment regimens at the species level for rare infections when it is possible. Repurposing an already licensed, well tolerated, readily available antimicrobial drug with excellent tissue distribution properties such as doxycycline might be advantageous for *M chimaera,* as drug tissue penetration is important and drug discovery funding is not a priority.

MICs were established in this study using the CLSI microbroth dilution method^13^ with modifications to the media, incorporating the commonly used mycobacterial media Middlebrook 7H9 (ADC) with 0·05% Tween 80 detergent in place of Mueller Hinton Broth (Middlebrook OADC supplement) media. The use of this media enhanced the reproducibility of the assays by reducing mycobacterial clumping; which also minimised the impact of drug tolerance arising from bacterial clumping, and it enabled the comparison of drug susceptibility between the three mycobacteria. However, it should be noted that such changes to the media can influence mycobacterial physiology through alternative carbon sources and cell wall composition, and these changes could alter antimicrobial drug efficacy. Better whole cell screening models are needed that are more representative of real-world events, which reproduce the in-vivo environments where the antimicrobial drugs act. This study focused on three mycobacterial reference strains: *M chimaera* NCTC13781, *M abscessus* ATCC19977, and *M tuberculosis* H37Rv. Further exploration of drug repurposing options on a range of clinical isolates will be essential due to the wide distribution of antimicrobial drug MICs found across non-tuberculous mycobacteria.

This study is limited because it only demonstrates in-vitro drug efficacies against an *M chimaera* reference strain; and antimicrobial drug susceptibility testing in vitro does not always correlate with in-vivo activity. Griffith and Winthrop recently discussed the challenges of treating non-tuberculous mycobacteria: low MIC values might not correlate to clinical efficacy, clinical isolates might exhibit a broad range of MICs with no clear demarcation between susceptible and resistant isolates, and isolates might have high MIC values but no readily identifiable resistance mechanism.^25^ Despite these caveats, in-vitro susceptibility testing still has merit in research and clinical diagnostic settings.^26^ This is especially true for non-tuberculous mycobacteria, in which in-vitro drug efficacy data is incomplete and the scope for clinical trials to optimise treatments is restricted.^27^

This is an exploratory study; therefore, further work confirming antimicrobial drug activity against *M chimaera* clinical isolates, the demonstration of in-vivo efficacy, and clinical validation of the observations in this study is required before these findings influence clinical practice. However, we identify the diaminoquinazoline, MMV675968, as an antimicrobial compound with 2 μM activity against *M chimaera*, highlighting folate metabolism as a druggable pathway in this bacterium. In addition, we suggest drug repurposing opportunities for doxycycline, bedaquiline, and oxazolidinones in the treatment of rare but often fatal *M chimaera* disease.

## Data sharing

The Pathogen Box screening results for all the compounds are detailed in the appendix, and they are deposited in the ChEMBL database (https://www.ebi.ac.uk/chembl/) (ChEMBL ID: CHEMBL4665541).

## Declaration of interests

We declare no competing interests.

## References

[1] Kohler P, Kuster SP, Bloemberg G (2015). Healthcare-associated prosthetic heart valve, aortic vascular graft, and disseminated *Mycobacterium chimaera* infections subsequent to open heart surgery. Eur Heart J.

[2] van Ingen J, Kohl TA, Kranzer K (2017). Global outbreak of severe *Mycobacterium chimaera* disease after cardiac surgery: a molecular epidemiological study. Lancet Infect Dis.

[3] Scriven JE, Scobie A, Verlander NQ (2018). *Mycobacterium chimaera* infection following cardiac surgery in the UK: clinical features and outcome of the first 30 cases. Clin Microbiol Infect.

[4] Larcher R, Lounnas M, Dumont Y (2019). *Mycobacterium chimaera* pulmonary disease in cystic fibrosis patients, France, 2010–17. Emerg Infect Dis.

[5] Schweickert B, Goldenberg O, Richter E (2008). Occurrence and clinical relevance of *Mycobacterium chimaera* sp nov, Germany. Emerg Infect Dis.

[6] Tan N, Sampath R, Abu Saleh OM (2016). Disseminated *Mycobacterium chimaera* infection after cardiothoracic surgery. Open Forum Infect Dis.

[7] Daley CL, Iaccarino JM, Lange C (2020). Treatment of nontuberculous mycobacterial pulmonary disease: an official ATS/ERS/ESCMID/IDSA clinical practice guideline. Eur Respir J.

[8] Public Health England (2017). *Mycobacterium chimaera* infections associated with cardiopulmonary bypass. https://www.gov.uk/government/publications/mycobacterium-chimaera-infections-guidance-for-secondary-care.

[9] Maurer FP, Pohle P, Kernbach M (2019). Differential drug susceptibility patterns of *Mycobacterium chimaera* and other members of the *Mycobacterium avium*-intracellulare complex. Clin Microbiol Infect.

[10] Schulthess B, Schäfle D, Kälin N, Widmer T, Sander P (2021). Drug susceptibility distributions of *Mycobacterium chimaera* and other non-tuberculous mycobacteria. Antimicrob Agents Chemother.

[11] Palomino JC, Martin A, Camacho M, Guerra H, Swings J, Portaels F (2002). Resazurin microtiter assay plate: simple and inexpensive method for detection of drug resistance in *Mycobacterium tuberculosis*. Antimicrob Agents Chemother.

[12] Medicines for Malaria Venture (2019). About the Pathogen Box. https://www.mmv.org/mmv-open/pathogen-box/about-pathogen-box.

[13] Woods GL, Brown-Elliott BA, Conville PS (2018).

[14] Zhang JH, Chung TD, Oldenburg KR (1999). A simple statistical parameter for use in evaluation and validation of high throughput screening assays. J Biomol Screen.

[15] Yan Ling JR, David Zhang, Ben Gold, Carl Nathan Pathogen Box Activity, Biological Data Structures. Medicines for Malaria Venture. https://www.mmv.org/sites/default/files/uploads/docs/mmv_open/Pathogen_Box_Activity_Biological_Data_Smiles.xlsx.

[16] Haworth CS, Banks J, Capstick T (2017). British Thoracic Society guidelines for the management of non-tuberculous mycobacterial pulmonary disease (NTM-PD). Thorax.

[17] Songsungthong W, Yongkiettrakul S, Bohan LE (2019). Diaminoquinazoline MMV675968 from pathogen box inhibits *Acinetobacter baumannii* growth through targeting of dihydrofolate reductase. Sci Rep.

[18] Zheng J, Rubin EJ, Bifani P (2013). Para-aminosalicylic acid is a prodrug targeting dihydrofolate reductase in *Mycobacterium tuberculosis*. J Biol Chem.

[19] Duffy S, Sykes ML, Jones AJ (2017). Screening the medicines for malaria venture pathogen box across multiple pathogens reclassifies starting points for open-source drug discovery. Antimicrob Agents Chemother.

[20] Gatto GJ, Ao Z, Kearse MG (2013). NADPH oxidase-dependent and oxidase-independent mechanisms of reported inhibitors of reactive oxygen generation. J Enzyme Inhib Med Chem.

[21] Low JL, Wu ML, Aziz DB, Laleu B, Dick T (2017). Screening of TB actives for activity against nontuberculous mycobacteria delivers high hit rates. Front Microbiol.

[22] Martin A, Godino IT, Aguilar-Ayala DA, Mathys V, Lounis N, Villalobos HR (2019). In vitro activity of bedaquiline against slow-growing nontuberculous mycobacteria. J Med Microbiol.

[23] Ruth MM, Sangen JJN, Remmers K (2019). A bedaquiline-clofazimine combination regimen might add activity to the treatment of clinically relevant non-tuberculous mycobacteria. J Antimicrob Chemother.

[24] Philley JV, Wallace RJ, Benwill JL (2015). Preliminary results of bedaquiline as salvage therapy for patients with nontuberculous mycobacterial lung disease. Chest.

[25] Griffith DE, Winthrop KL (2021). You gotta make me see, what does it mean to have an MIC?. Chest.

[26] Mok S, Hannan MM, Nölke L (2019). Antimicrobial susceptibility of clinical and environmental *Mycobacterium chimaera* isolates. Antimicrob Agents Chemother.

[27] Lee SFK, Laughon BE, McHugh TD, Lipman M (2019). New drugs to treat difficult tuberculous and nontuberculous mycobacterial pulmonary disease. Curr Opin Pulm Med.

