# Supplementary material for: Searching for new therapeutic options for the uncommon pathogen Mycobacterium chimaera: an open drug discovery approach
Source: Lancet Microbe. 2022 May;3(5):e382–91. doi: 10.1016/S2666-5247(21)00326-8 (PMC9042791; doi:10.1016/S2666-5247(21)00326-8)
Supplement: Supplementary appendix [file mmc1.pdf]

THE LANCET
Microbe

Supplementary appendix

This appendix formed part of the original submission and has been peer reviewed. We post it as supplied by the authors.

Supplement to: Cantillon D, Goff A, Taylor S, et al. Searching for new therapeutic options for the uncommon pathogen *Mycobacterium chimaera*: an open drug discovery approach. *Lancet Microbe* 2022; published online April 1. https://doi.org/10.1016/S2666-5247(21)00326-8.

Supplementary Appendix

Table of Contents

1. Figure S1: Overview of the *M.chimaera* Pathogen Box screen.....……………………..……………………..2
2. Figure S2: Flowchart describing MIC determination methodologies…...………………………………....3
3. Figure S3: Comparison of *Low et al*. and this study *M.abscessus* Pathogen Box hits………..……........4
4. Figure S4: Comparison of *Low et al*. *M.avium* hits with *M.chimaera* hits from this study……..……..…...5
5. Table S1: Screening results of all Pathogen Box compounds against *M.chimaera*, *M.tuberculosis* and *M.abscessus*………...……………………………………………………………..………………...7
6. Table S2: Physicochemical properties of four hit compounds with MICs determined against *M.chimaera*……………………………………………………………………………..……….…….….17
7. Table S3: Cytotoxicity and drug metabolism pharmacokinetics data of four hit compounds with MICs determined against *M.chimaera*…………………………………………………….………………18
8. References ………………………………………………………………………………………..........19

Figure S1: Overview of the *M.chimaera* Pathogen Box screen.

M.chimaera reference strain (NCTC13781) was screened against the Medicines for Malaria Venture Pathogen Box.[1] 1) Log phase *M.chimaera* was diluted to ~10^5 CFU/mL based on optical density and added to microtitre plates containing the Pathogen Box compound library at a final concentration of 20 µM (2% DMSO). 2) Pathogen Box microtitre plates were incubated for seven days at 37°C before adding CellTiter Blue. Following 16h incubation, viable bacteria convert the blue resazurin in CellTiter Blue to the pink resorufin. This blue to pink conversion correlates with cell viability. 3) Fluorescence was quantified using a plate reader, and % growth inhibition determined in comparison to compound-free positive growth control wells. 4) Selected hits were taken forward for determination of MIC, and time kill kinetics. Created with BioRender.com.

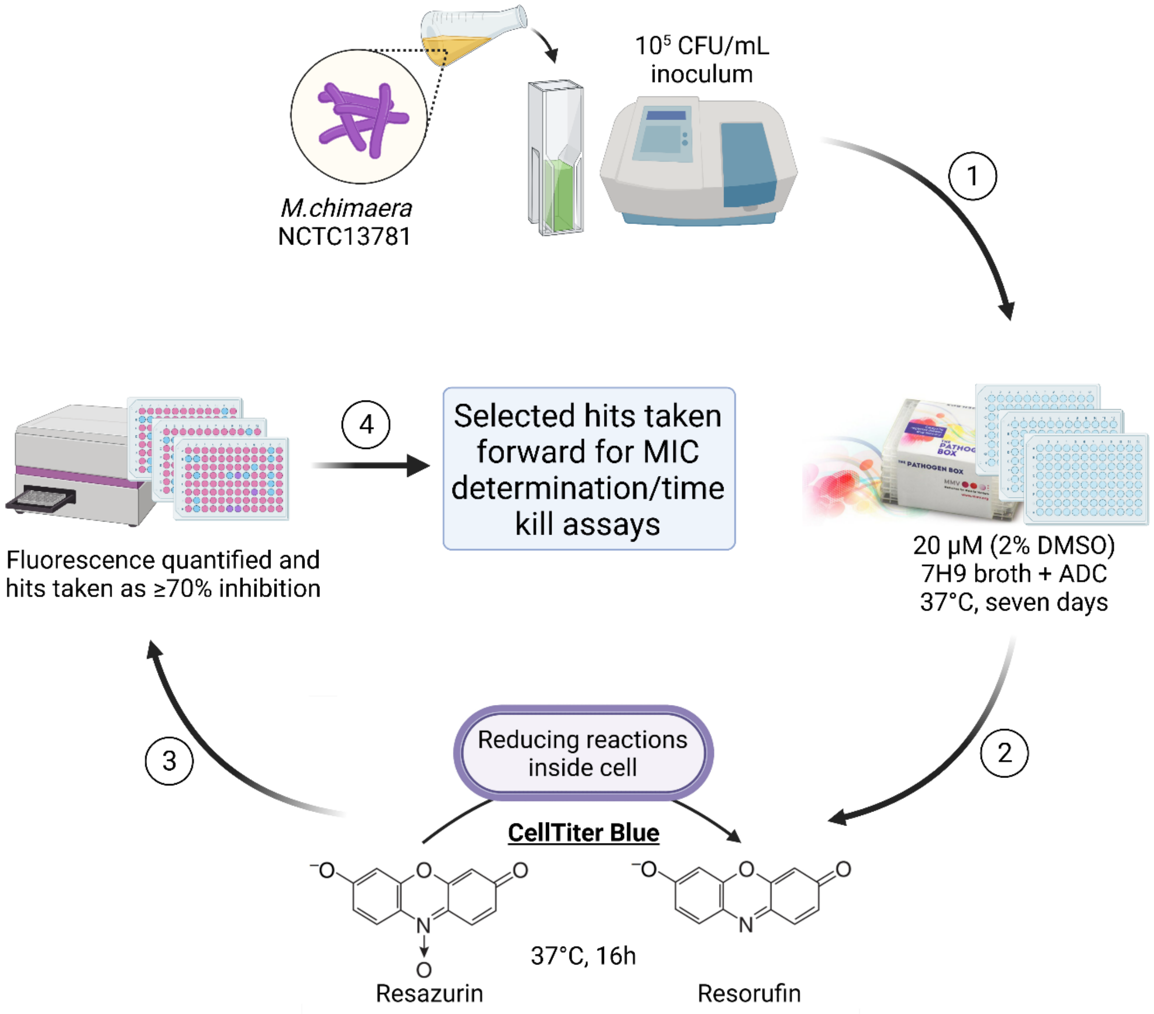

Figure S2: Flowchart describing MIC determination methodologies.

Comparison of microbroth dilution methods to determine antimicrobial drug MICs in NTM. Clinical and Laboratory Standards Institute (CLSI) methodology[2] detailed in blue, alongside modified protocol used in this study (in green). Created with BioRender.com.

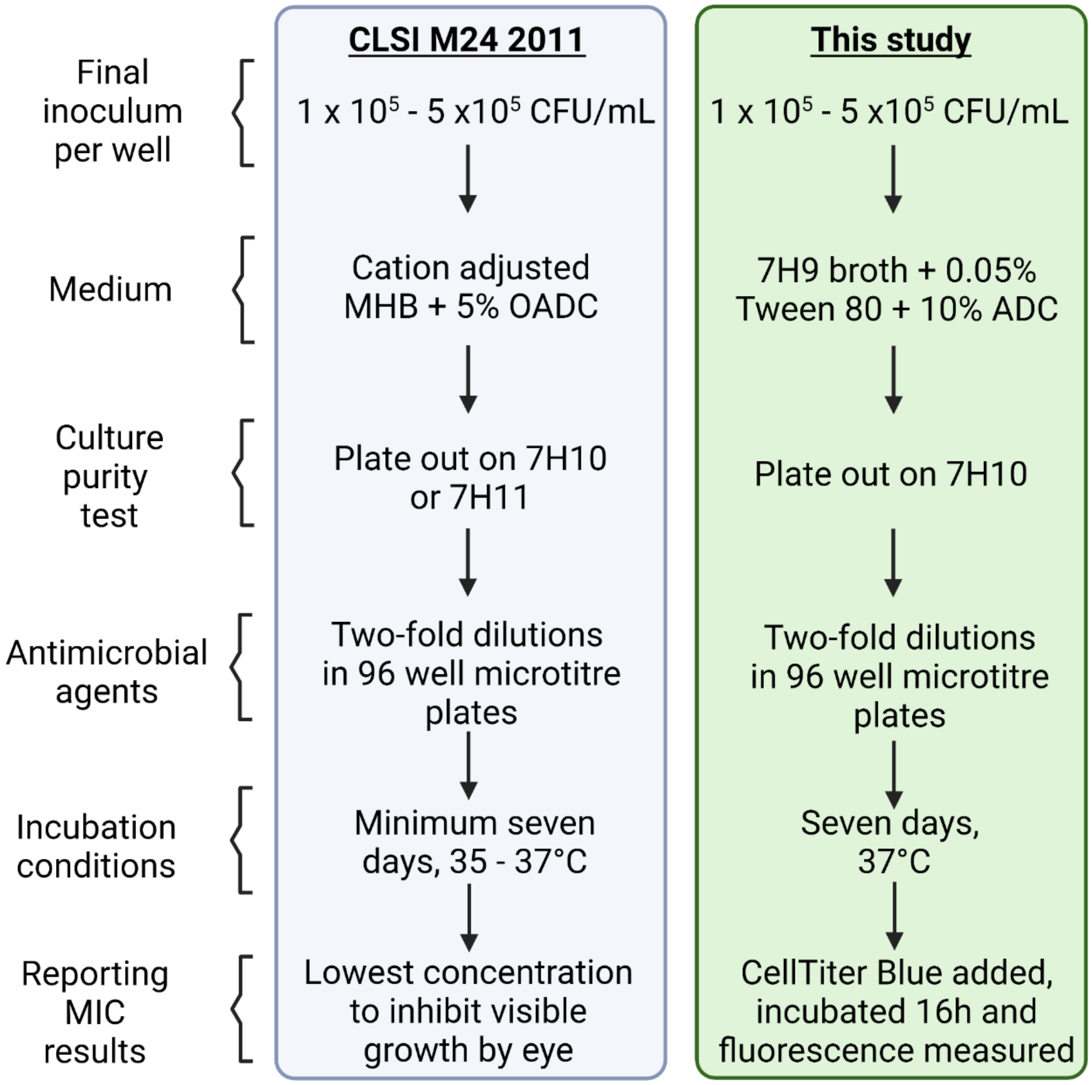

Figure S3: Comparison of Low *et al.* and this study *M.abscessus* Pathogen Box hits.

(A) The Pathogen Box was screened against an *M.abscessus* clinical isolate[3] and against *M.abscessus* ATCC19977 (this study), where hits were classified as ≤ 20% survival and ≤ 30% survival respectively. Two compounds were hits in Low *et al*. but not in this study (marked in A), while three compounds were hits in this study but not in Low *et al.* (marked in A). Overall there was good agreement between screening results. (B) Details of the 11 compounds classed as hits in both *M.abscessus* screens.

(A)

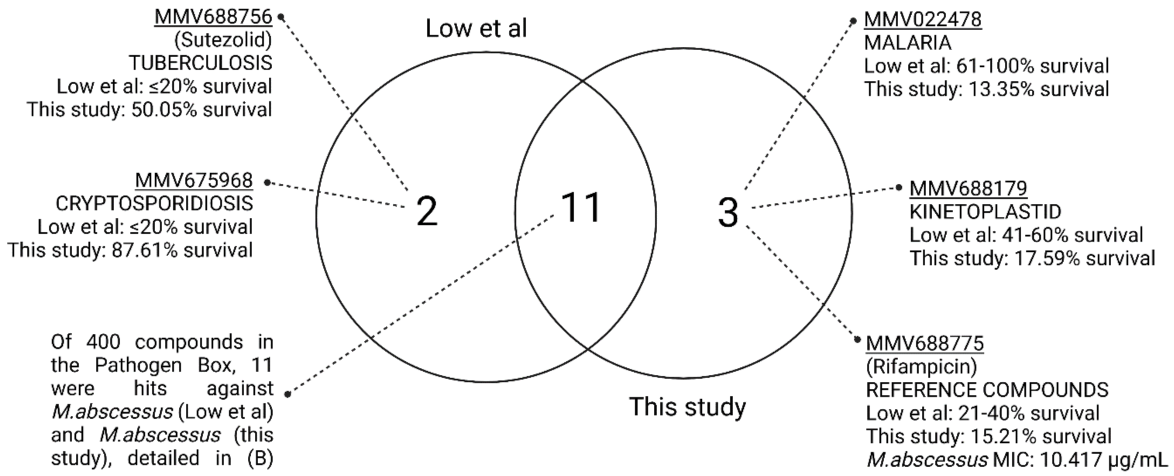

(B)

MMV number	Class	Trivial Name	*M.abscessus* % Survival (Low *et al.*[3])	*M.abscessus* % Survival (this study)
MMV689758	Reference compounds	Bedaquiline	≤20	19·76
MMV687803	Reference compounds	Linezolid	≤20	24·52
MMV688508	Tuberculosis	-	≤20	18·57
MMV687730	Tuberculosis	-	≤20	18·76
MMV688845	Tuberculosis	-	≤20	24·1
MMV687146	Tuberculosis	-	≤20	17·24
MMV687798	Reference compounds	Levofloxacin	≤20	11·47
MMV688327	Tuberculosis	Radezolid	≤20	18·57
MMV688846	Tuberculosis	-	≤20	20·92
MMV688844	Tuberculosis	-	≤20	11·71
MMV687812	Tuberculosis	-	≤20	26·85

Figure S4: Comparison of Low *et al.* *M.avium* hits with *M.chimaera* hits from this study.

(A) The Pathogen Box was screened against an *M.avium* clinical isolate[3] and against *M.chimaera* NCTC13781 (this study), where antimicrobial hits were classified as ≤ 20% survival and ≤ 30% survival respectively. Four compounds were classified as hits against *M.chimaera* in this study that were not identified as hits against *M.avium* in Low *et al*. (marked in A). (B) Details of 16 compounds that were hits against *M.avium* but not *M.chimaera*. (C) Details of 17 compounds that were classed as hits in both studies.

(A)

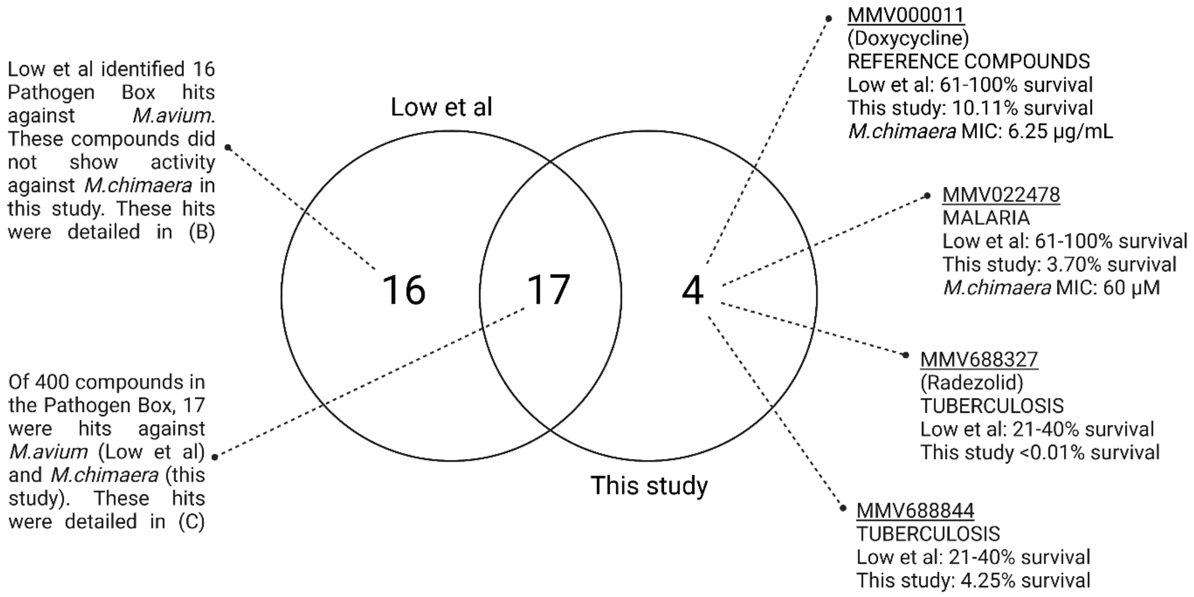

(B)

MMV number	Class	Trivial Name	*M.avium* % Survival (Low *et al.*[3])	*M.chimaera* % Survival (this study)
MMV054312	Tuberculosis	-	≤20	76·76
MMV661713	Tuberculosis	-	≤20	47·45
MMV688994	Reference compounds	Streptomycin	≤20	80·03
MMV000062	Reference compounds	Pentamidine	≤20	85·25
MMV688262	Tuberculosis	Delaminid	≤20	112·57
MMV687145	Tuberculosis	-	≤20	129·84
MMV063404	Tuberculosis	-	≤20	90·41
MMV687730	Tuberculosis	-	≤20	87·35
MMV676383	Tuberculosis	-	≤20	79·30
MMV676377	Tuberculosis	-	≤20	77·67
MMV687696	Tuberculosis	-	≤20	75·71
MMV012074	Tuberculosis	-	≤20	88·40
MMV687813	Tuberculosis	-	≤20	34·39
MMV687188	Tuberculosis	-	≤20	73·54
MMV000014	Reference compounds	Mefloquine	≤20	109·85
MMV687807	Tuberculosis	-	≤20	82·94

(C)

**MMV number**	**Class**	**Trivial Name**	***M.avium* % Survival (Low *et al.*[3])**	***M.chimaera* % Survival (this study)**
MMV687146	Tuberculosis	-	≤20	4·50
MMV688845	Tuberculosis	-	≤20	1·95
MMV153413	Tuberculosis	-	≤20	9·37
MMV689758	Tuberculosis	-	≤20	1·81
MMV676395	Tuberculosis	-	≤20	-0·21
MMV687798	Reference compounds	Levofloxacin	≤20	1·47
MMV688775	Reference compounds	Rifampicin	≤20	1·46
MMV688271	Kinetoplastids	-	≤20	3·73
MMV675968	Tuberculosis	-	≤20	4·50
MMV688508	Tuberculosis	-	≤20	-0·22
MMV687703	Tuberculosis	-	≤20	3·71
MMV687729	Tuberculosis	-	≤20	30·51
MMV687800	Reference compounds	Clofazimine	≤20	6·51
MMV688756	Tuberculosis	Sutezolid	≤20	2·59
MMV461553	Tuberculosis	-	≤20	7·21
MMV688179	Kinetoplastids		≤20	2·34
MMV687803	Reference compounds	Linezolid	≤20	10·81

Table S1: Screening results of all Pathogen Box compounds against *M.chimaera*, *M.tuberculosis* and *M.abscessus*.

Detailing % survival for the three pathogens compared to drug-free positive controls, alongside Pathogen Box rack and plate position, MMV number, drug classification and formula. The hits identified in this study for each mycobacterium are marked in the final three columns.

Rack	Position	MMV number	Class	Trivial Name	Molecular Formula	*M. chimaera* % survival	*M. tuberculosis* % survival	*M. abscessus* % survival	*M. chimaera* hit	*M. tuberculosis* hit	*M. abscessus* hit
PlateB	E04	MMV000011	REFERENCE COMPOUNDS	Doxycycline	C22H24N2O8	10·11	22·44	117·83	YES	YES	
PlateB	H03	MMV000016	REFERENCE COMPOUNDS	Mefloquine	C17H16N2OF6	109·85	126·64	99·94			
PlateB	G11	MMV000023	REFERENCE COMPOUNDS	Primaquine	C15H21N3O	92·82	84·68	109·23			
PlateB	A04	MMV000062	REFERENCE COMPOUNDS	Pentamidine	C19H24N4O2	85·25	18·83	109·30		YES	
PlateB	D05	MMV000063	REFERENCE COMPOUNDS	Sitamaquine	C21H33N3O	99·84	90·61	119·50			
PlateB	G08	MMV000858	MALARIA		C16H18N3O3ClS2	118·45	105·19	113·02			
PlateA	B03	MMV000907	MALARIA		C11H13N5O	98·30	97·11	111·26			
PlateD	G03	MMV001059	MALARIA		C17H24NO3F3S	105·58	89·40	105·14			
PlateB	C06	MMV001493	ONCHOCERCIASIS	Isradipine	C19H21N3O5	105·50	93·59	127·72			
PlateE	D06	MMV001499	REFERENCE COMPOUNDS	Nifurtimox	C10H13N3O5S	100·92	93·17	125·26			
PlateE	G07	MMV001561	KINETOPLASTIDS	Fluoxetine	C17H18NOF3	102·84	91·31	102·83			
PlateB	C05	MMV001625	REFERENCE COMPOUNDS	alpha-Difluoromethylornithine	C6H12N2O2F2	100·74	90·48	120·98			
PlateB	B04	MMV002529	REFERENCE COMPOUNDS	Praziquantel	C19H24N2O2	99·77	91·98	120·07			
PlateE	E03	MMV002816	REFERENCE COMPOUNDS	Diethylcarbamazine	C10H21N3O	101·60	86·42	112·29			
PlateB	E06	MMV002817	ONCHOCERCIASIS	Iodoquinol	C9H5NOI2	100·08	90·57	118·21			
PlateB	G06	MMV003152	REFERENCE COMPOUNDS	Mebendazole	C16H13N3O3	96·65	92·67	111·35			
PlateB	F04	MMV003270	HOOKWORM	Zoxazolamine	C7H5N2OCl	99·21	85·59	109·46			
PlateE	B06	MMV004168	KINETOPLASTIDS		C19H23N2Cl	99·65	90·16	113·97			
PlateB	G07	MMV006239	MALARIA		C18H14N3OBr	94·84	86·87	111·84			
PlateB	A05	MMV006372	MALARIA		C15H19N5	98·36	100·89	110·82			
PlateB	G09	MMV006741	MALARIA		C22H26N4O2	96·40	85·66	111·43			
PlateD	C03	MMV006833	MALARIA		C19H27N2O4ClS	114·68	93·72	125·77			
PlateB	H08	MMV006901	MALARIA		C18H18N4O	120·10	86·84	101·69			
PlateD	H02	MMV007133	MALARIA		C21H23N3O4S	95·16	98·74	94·96			
PlateD	B04	MMV007471	MALARIA		C23H21N2O4Cl	101·18	89·10	119·11			
PlateD	B03	MMV007625	MALARIA		C22H27N5OS	104·06	90·41	123·30			
PlateB	B10	MMV007638	MALARIA		C20H20N2O4S2	112·89	100·04	122·53			
PlateD	G02	MMV007803	MALARIA		C16H16NO2ClS	105·28	97·46	100·85			

PlateE	A09	MMV007920	MALARIA		C20H15N3O2	99·67	98·00	101·93			
PlateC	A09	MMV008439	MALARIA		C23H32N2O3	99·83	106·44	112·30			
PlateB	H07	MMV009054	MALARIA		C15H12N4O2Cl2	100·15	98·72	105·48			
PlateD	F02	MMV009135	MALARIA		C22H24N6O	103·60	93·60	101·03			
PlateE	D08	MMV010545	MALARIA		C19H18N6O3S	86·39	91·37	110·96			
PlateD	A05	MMV010576	MALARIA		C19H18N2O4S	98·99	106·47	112·13			
PlateA	A02	MMV010764	MALARIA		C14H16N4OS2	94·11	88·79	100·80			
PlateE	A02	MMV011229	MALARIA		C14H11N4F3	94·02	92·61	97·35			
PlateD	B02	MMV011511	MALARIA		C23H33N3O	103·88	87·99	112·09			
PlateD	G04	MMV011691	MALARIA		C21H21N5O3	102·69	86·17	104·45			
PlateD	F03	MMV011765	MALARIA		C13H6N4OClF3S	70·94	89·24	107·69			
PlateB	A07	MMV011903	MALARIA		C20H18N6O2	107·68	108·25	114·12			
PlateB	B02	MMV012074	TUBERCULOSIS		C16H14N3OCl	88·40	53·35	110·14			
PlateB	D10	MMV016136	MALARIA		C19H16N5Cl	108·25	107·40	118·40			
PlateE	F04	MMV016838	MALARIA		C22H16N3OBr	91·69	78·88	108·85			
PlateB	F08	MMV019087	MALARIA		C19H19N6O2F3	92·65	74·93	111·31			
PlateB	F02	MMV019189	MALARIA		C17H15N3OS	97·40	76·96	97·21			
PlateB	D09	MMV019234	MALARIA		C20H18N2O4S	92·92	78·09	124·65			
PlateE	F02	MMV019551	MALARIA		C20H22N2O4	90·10	77·84	102·62			
PlateB	C10	MMV019721	MALARIA		C19H20N3O3ClS2	113·96	103·57	120·90			
PlateB	H06	MMV019742	MALARIA		C21H22N4O3S	97·46	96·00	103·39			
PlateD	E11	MMV019790	MALARIA		C22H20N4O2	101·84	90·16	107·36			
PlateB	E10	MMV019807	MALARIA		C18H14N3OCl	118·28	97·44	115·67			
PlateB	D07	MMV019838	MALARIA		C18H10N4OF6	81·16	84·17	124·12			
PlateE	A11	MMV019993	MALARIA		C17H17N6F3	98·93	97·05	95·92			
PlateD	D03	MMV020081	MALARIA		C22H24N3O2F	87·74	89·42	117·73			
PlateB	B08	MMV020120	MALARIA		C23H34N2O	104·03	94·27	122·38			
PlateB	C07	MMV020136	MALARIA		C15H18N4O3S2	88·55	88·67	128·71			
PlateB	E08	MMV020152	MALARIA		C17H14N2O2	91·20	78·23	117·76			
PlateE	C09	MMV020165	MALARIA		C19H20N3O2F3	98·69	82·07	105·66			
PlateE	E02	MMV020289	MALARIA		C18H23N3O3S	102·96	86·32	112·86			
PlateD	C02	MMV020291	MALARIA		C21H33N3O3S	102·74	90·68	120·57			
PlateB	F10	MMV020320	MALARIA		C20H17N5O2	98·86	42·16	112·60			
PlateB	F07	MMV020321	MALARIA		C18H18N2O2ClF3	91·65	90·70	115·48			
PlateC	B02	MMV020388	MALARIA		C19H28N3O2Cl	101·84	88·49	112·68			
PlateB	H09	MMV020391	MALARIA		C19H23N2O3ClS	91·79	95·44	107·67			
PlateB	A10	MMV020512	MALARIA		C25H25N3O3	109·21	103·67	116·09			
PlateB	C09	MMV020517	MALARIA		C21H24N4O2	94·05	79·94	123·40			
PlateB	D08	MMV020520	MALARIA		C25H27N3O3	113·20	103·75	124·81			
PlateB	D02	MMV020537	MALARIA		C16H15N2O2ClS	62·74	94·09	105·44			
PlateB	A08	MMV020591	MALARIA		C24H23N3O2	111·66	106·12	113·36			
PlateB	A09	MMV020623	MALARIA		C25H26N4O2	110·64	107·26	117·87			
PlateD	A03	MMV020670	MALARIA		C22H21N5O2	93·02	99·72	109·80			
PlateB	C08	MMV020710	MALARIA		C19H18N5O3Cl	95·67	86·94	123·14			
PlateB	B07	MMV020982	MALARIA		C22H22N4O2	102·56	84·27	123·59			
PlateE	G04	MMV021013	TUBERCULOSIS		C18H22N4	88·04	100·06	108·06			

PlateB	B11	MMV021057	MALARIA	Azoxystrobin	C22H17N3O5	110·15	96·64	125·90			
PlateC	E10	MMV021375	MALARIA		C17H20N4	93·83	89·56	111·09			
PlateC	G04	MMV021660	TUBERCULOSIS		C20H21N6Cl	106·97	67·49	101·99			
PlateD	B07	MMV022029	MALARIA		C26H31N3O2S	99·10	90·76	117·88			
PlateC	C11	MMV022236	MALARIA		C21H28N5O2F3	109·38	109·06	119·04			
PlateD	H03	MMV022478	MALARIA		C23H21N6OCl	3·70	3·81	13·35	YES	YES	YES
PlateE	B08	MMV023183	MALARIA		C25H30N4O	103·66	88·35	119·06			
PlateE	D09	MMV023227	MALARIA		C18H19N3	87·20	85·27	105·84			
PlateD	E02	MMV023233	MALARIA		C16H12N4	90·81	89·11	105·98			
PlateC	G10	MMV023370	MALARIA		C14H10N7Cl	106·93	102·35	108·74			
PlateD	H08	MMV023388	MALARIA		C21H18N3OF3	98·01	99·29	90·41			
PlateD	D07	MMV023860	MALARIA		C18H18N4O3S	90·29	84·57	117·18			
PlateD	D09	MMV023949	MALARIA		C24H27N7O	90·52	92·68	116·06			
PlateD	A04	MMV023953	MALARIA		C25H28N4O	93·72	99·99	104·45			
PlateC	F04	MMV023969	TUBERCULOSIS		C24H24N4OS	92·85	19·47	95·96		YES	
PlateD	F06	MMV023985	MALARIA		C18H20N4O2	108·64	85·08	100·89			
PlateD	B10	MMV024035	MALARIA		C25H23N3O2S	98·30	85·90	117·22			
PlateD	H04	MMV024101	MALARIA		C16H12N6O2S	100·09	99·59	95·79			
PlateD	C07	MMV024114	MALARIA		C19H15N4O2BrS	106·11	125·65	121·30			
PlateD	F07	MMV024195	MALARIA		C22H28N6OS	94·13	74·23	103·32			
PlateC	H07	MMV024311	TUBERCULOSIS		C19H21N3	105·51	74·43	96·57			
PlateB	E09	MMV024397	MALARIA		C21H25N3O	98·18	88·82	117·57			
PlateD	D11	MMV024406	MALARIA		C21H19N5OCl2	100·11	89·04	114·31			
PlateD	H06	MMV024443	MALARIA		C20H16N4O	101·42	101·61	94·48			
PlateD	B05	MMV024829	MALARIA		C23H32N4O	102·83	88·91	119·19			
PlateD	F04	MMV024937	MALARIA		C20H18N5O2F3	111·19	88·43	103·25			
PlateA	F02	MMV026020	MALARIA		C23H20N2O4S	104·99	102·30	113·07			
PlateE	F09	MMV026313	MALARIA		C14H11N3O	86·22	82·90	100·47			
PlateD	A11	MMV026356	MALARIA		C20H27N7O2	89·72	101·08	106·63			
PlateD	A02	MMV026468	MALARIA		C18H15N2OCl	96·11	95·93	105·25			
PlateD	C04	MMV026490	MALARIA		C23H24N4O	104·42	88·91	115·44			
PlateD	D04	MMV026550	MALARIA		C20H23N3O	98·69	88·20	111·25			
PlateE	D05	MMV028694	MALARIA		C18H16N6O	91·38	88·74	113·04			
PlateE	E05	MMV030734	MALARIA		C17H19N6OCl	89·37	82·63	111·00			
PlateD	A07	MMV031011	MALARIA		C25H29N4OCl	89·39	99·38	109·29			
PlateD	A06	MMV032967	MALARIA		C19H14N5F3	87·81	92·60	103·76			
PlateD	E08	MMV032995	MALARIA		C17H13NO2BrF3	98·37	89·12	110·60			
PlateD	B06	MMV045105	KINETOPLASTIDS		C19H19N3O2	101·58	106·67	113·33			
PlateE	B09	MMV047015	TUBERCULOSIS		C12H11N2OF3	85·29	52·24	110·64			
PlateA	C07	MMV053220	TUBERCULOSIS		C15H12N2O2	97·87	90·56	114·64			
PlateC	B07	MMV054312	TUBERCULOSIS		C15H16N2O	76·76	46·75	110·78			
PlateC	F10	MMV062221	MALARIA		C16H17N5	111·86	102·91	113·85			
PlateA	G06	MMV063404	TUBERCULOSIS		C19H24N3OCl	90·41	33·48	108·47			
PlateB	G02	MMV069458	TUBERCULOSIS		C6H2N4ClF3	94·07	11·93	101·88		YES	
PlateA	C02	MMV084603	MALARIA		C22H23N3OS	81·62	98·85	112·37			
PlateE	G09	MMV084864	MALARIA		C17H12N6O	100·40	84·16	100·75			

PlateD	E04	MMV085071	MALARIA		C19H20N6O	86·38	93·47	109·84			
PlateB	F11	MMV085210	MALARIA		C22H24N3O3ClS	88·16	82·81	114·57			
PlateD	E03	MMV085230	MALARIA		C18H24N2O3	110·77	93·73	117·66			
PlateD	F05	MMV085499	MALARIA		C18H13N5O	112·10	90·80	105·87			
PlateA	E07	MMV090930	TUBERCULOSIS		C16H13N3O4S2	84·64	38·64	109·62			
PlateA	H04	MMV099637	KINETOPLASTIDS		C17H16N3OF	95·29	108·90	98·36			
PlateC	H08	MMV1019989	MALARIA		C18H28N6	107·22	105·12	99·03			
PlateA	B10	MMV102872	TUBERCULOSIS		C16H9N3O2ClF3	97·68	18·32	114·19		YES	
PlateA	D03	MMV1028806	MALARIA		C18H14N3OFS	100·14	26·66	115·65		YES	
PlateC	E11	MMV1029203	MALARIA		C20H17N5OS	124·77	128·46	115·52			
PlateC	D11	MMV1030799	MALARIA		C20H18N4O	99·66	18·80	121·39		YES	
PlateC	H09	MMV1037162	MALARIA		C18H21N5OS	106·81	106·51	97·52			
PlateC	G08	MMV1088520	MALARIA		C18H21N3OS	98·24	102·28	109·76			
PlateA	B02	MMV1110498	WOLBACHIA LF		C15H16N3O2F3	101·51	97·94	110·70			
PlateC	H06	MMV1198433	SCHISTOSOMIASIS		C19H21N3O3	111·67	104·79	98·58			
PlateE	H09	MMV1236379	KINETOPLASTIDS		C12H12N2O3	98·41	96·03	110·25			
PlateE	G02	MMV146306	TUBERCULOSIS		C23H28N2O2	96·18	96·11	106·74			
PlateE	E11	MMV153413	TUBERCULOSIS		C16H11N2O4F5S	9·37	26·50	111·07	YES	YES	
PlateE	F10	MMV161996	TUBERCULOSIS		C20H25N2O3ClS	108·28	118·78	102·69			
PlateA	G04	MMV188296	KINETOPLASTIDS		C18H17N2O2F	97·99	94·98	108·57			
PlateE	D03	MMV200748	TUBERCULOSIS		C19H20NO2Br	100·48	103·36	108·42			
PlateA	H07	MMV202458	TUBERCULOSIS		C17H18NO3Cl	98·06	132·39	99·86			
PlateA	F05	MMV202553	KINETOPLASTIDS		C15H15N3O2	100·10	83·43	108·75			
PlateE	F07	MMV228911	TUBERCULOSIS		C21H19N3O3S	89·47	114·31	97·17			
PlateE	F08	MMV272144	TUBERCULOSIS		C9H10N4O3S	104·52	85·76	110·32			
PlateE	G05	MMV392832	MALARIA		C19H16N4O	93·80	75·42	105·98			
PlateE	A08	MMV393144	MALARIA		C18H19N4F	85·59	92·64	103·19			
PlateE	H08	MMV393995	TUBERCULOSIS		C10H12N4O	95·80	46·23	93·87			
PlateD	G07	MMV407539	WOLBACHIA LF		C24H24N4O3S	98·93	85·30	103·10			
PlateE	E09	MMV407834	MALARIA		C22H26N3O3F3	114·38	97·04	113·26			
PlateA	H09	MMV461553	TUBERCULOSIS		C18H14NO3F5S	7·21	42·18	103·00	YES		
PlateE	H11	MMV495543	TUBERCULOSIS		C14H16N2O2S	86·88	85·31	84·24			
PlateA	E04	MMV553002	TUBERCULOSIS		C10H9NO4S	66·36	34·76	112·65			
PlateB	E11	MMV560185	MALARIA		C19H19N3	89·93	80·75	115·86			
PlateC	A10	MMV595321	KINETOPLASTIDS		C21H26N6O4S	98·16	25·43	116·92		YES	
PlateE	C11	MMV611037	TUBERCULOSIS		C14H14N4O2S2	105·13	92·73	106·23			
PlateE	E04	MMV634140	MALARIA		C22H30N5O2Cl	83·98	80·99	110·39			
PlateB	F05	MMV637229	TRICHURIASIS	Clemastine	C21H26NOCl	96·43	86·97	112·15			
PlateB	D03	MMV637953	REFERENCE COMPOUNDS	Suramin	C51H40N6O23S6	117·22	102·46	145·46			
PlateB	A03	MMV652003	KINETOPLASTIDS		C15H11NO3BF3	97·17	112·01	109·34			
PlateE	G08	MMV658988	KINETOPLASTIDS		C19H19N4Cl	74·86	4·93	101·43		YES	
PlateD	G09	MMV658993	KINETOPLASTIDS		C19H19N4O2ClS	100·02	83·93	100·96			
PlateD	E05	MMV659004	KINETOPLASTIDS		C21H21N4Cl	100·02	6·84	111·91		YES	
PlateD	C10	MMV659010	KINETOPLASTIDS		C18H15N4F3	91·68	85·57	112·05			
PlateA	D04	MMV661713	TUBERCULOSIS		C21H19N6Br	47·45	25·17	112·44		YES	

PlateD	G06	MMV663250	MALARIA		C21H23N4Cl	115·61	95·42	103·61			
PlateE	D04	MMV667494	MALARIA		C25H34N5OClF2	95·66	84·12	110·50			
PlateB	H05	MMV668727	ONCHOCERCIASIS		C17H10N4F6	96·76	100·15	101·72			
PlateE	C04	MMV671636	ONCHOCERCIASIS		C23H14NO3F5	85·93	95·88	111·68			
PlateD	H09	MMV675968	CRYPTOSPORIDIOSIS		C17H18N5O2Cl	1·74	15·04	87·61	YES	YES	
PlateC	H02	MMV675969	ONCHOCERCIASIS		C21H28N2O2	106·92	95·79	93·65			
PlateC	G03	MMV675993	CRYPTOSPORIDIOSIS		C16H19N5O2	108·52	92·88	102·10			
PlateC	E02	MMV675994	CRYPTOSPORIDIOSIS		C18H13N4OCl	107·50	81·88	105·10			
PlateD	D05	MMV675995	ONCHOCERCIASIS		C17H20N2O2	97·40	90·77	111·00			
PlateD	H10	MMV675996	ONCHOCERCIASIS		C22H28N2O2	94·31	101·57	94·23			
PlateC	A02	MMV675997	KINETOPLASTIDS		C24H29N4O2F	98·51	99·41	105·02			
PlateD	C09	MMV675998	KINETOPLASTIDS		C17H17N7O	94·69	89·70	117·44			
PlateD	C11	MMV676008	KINETOPLASTIDS		C24H15N3O6	115·55	99·74	122·93			
PlateC	D03	MMV676048	KINETOPLASTIDS		C16H18N2OCl2	94·39	90·47	113·66			
PlateD	D10	MMV676050	CRYPTOSPORIDIOSIS		C23H26N6O2S	95·82	84·82	110·27			
PlateC	F02	MMV676053	CRYPTOSPORIDIOSIS		C18H16N3O3Cl	105·49	89·40	97·14			
PlateC	E03	MMV676057	KINETOPLASTIDS		C24H28N4O3	110·03	108·80	109·04			
PlateD	F08	MMV676063	ONCHOCERCIASIS		C30H36N3O2F	111·62	90·98	105·14			
PlateD	B08	MMV676064	ONCHOCERCIASIS		C23H21N2O2F	102·39	96·81	117·29			
PlateE	A07	MMV676159	KINETOPLASTIDS		C19H18N3OCl	100·43	99·40	105·66			
PlateE	B07	MMV676161	KINETOPLASTIDS		C20H21N3O2	102·71	80·18	113·97			
PlateD	C06	MMV676162	KINETOPLASTIDS		C21H14N2O4	112·45	93·29	117·04			
PlateD	G10	MMV676182	CRYPTOSPORIDIOSIS		C22H24N6	103·84	91·36	101·98			
PlateD	F09	MMV676186	KINETOPLASTIDS		C26H19N3O6	98·76	94·51	105·47			
PlateC	G02	MMV676191	CRYPTOSPORIDIOSIS		C15H16O3	105·34	99·97	99·49			
PlateC	A03	MMV676204	ONCHOCERCIASIS		C21H19N2OF	101·45	99·68	107·18			
PlateD	E06	MMV676260	MALARIA		C17H17N5F2	97·10	84·76	107·94			
PlateD	D02	MMV676269	MALARIA		C21H25N2O2F	103·80	88·84	110·53			
PlateE	F05	MMV676270	MALARIA		C21H25N2O2F	102·08	82·73	108·46			
PlateA	E03	MMV676350	MALARIA		C17H16N3OClS	104·83	94·93	116·28			
PlateE	E07	MMV676358	MALARIA		C11H7N5BrCl	92·32	98·44	113·69			
PlateA	F08	MMV676377	TUBERCULOSIS		C13H8N3O2Br	77·67	50·16	107·98			
PlateA	C11	MMV676379	TUBERCULOSIS		C15H13O3Br	87·94	98·00	115·72			
PlateB	H10	MMV676380	MALARIA		C18H15N4O3Cl	86·93	97·03	102·33			
PlateB	C04	MMV676382	SCHISTOSOMIASIS		C20H23N7O	98·96	88·39	121·74			
PlateA	D09	MMV676383	TUBERCULOSIS		C14H13N3O2S2	79·30	1·20	110·25		YES	
PlateE	D11	MMV676384	TUBERCULOSIS		C18H14N3OF	100·75	54·53	108·81			
PlateB	E02	MMV676386	TUBERCULOSIS		C19H17N3O3	90·14	23·98	107·49		YES	
PlateA	F04	MMV676388	TUBERCULOSIS		C15H14N4O3S	101·03	87·41	109·97			
PlateA	B07	MMV676389	TUBERCULOSIS		C20H24N4O3	98·65	51·70	113·28			
PlateA	C10	MMV676395	TUBERCULOSIS		C17H13N3O3	-0·21	2·49	110·74	YES	YES	
PlateE	C02	MMV676398	WOLBACHIA LF		C18H20N2O4S	99·27	92·05	110·71			
PlateA	B09	MMV676401	TUBERCULOSIS		C18H20N4O	87·98	95·80	112·69			
PlateA	F09	MMV676406	TUBERCULOSIS		C26H29N5O2S	104·90	67·79	110·70			
PlateA	D11	MMV676409	TUBERCULOSIS		C16H12N3O2FS	85·66	19·22	121·58		YES	
PlateD	G11	MMV676411	TUBERCULOSIS		C17H15N3O2S	98·22	16·36	101·21		YES	

PlateA	A11	MMV676412	TUBERCULOSIS		C16H9N2O3ClS	92·81	6·60	105·12		YES	
PlateA	E08	MMV676431	TUBERCULOSIS		C17H16NO3Cl	98·62	81·49	114·71			
PlateA	C09	MMV676439	TUBERCULOSIS		C16H15N2ClS	34·69	91·68	114·22			
PlateB	E07	MMV676442	MALARIA		C25H29N5O	97·11	86·76	122·57			
PlateA	D10	MMV676444	TUBERCULOSIS		C19H27N3O3	97·49	56·32	113·18			
PlateA	E10	MMV676445	TUBERCULOSIS		C17H19N3O	74·21	30·04	111·05		YES	
PlateA	A10	MMV676449	TUBERCULOSIS		C19H21N2O2Cl	84·78	90·18	101·71			
PlateA	F10	MMV676461	TUBERCULOSIS		C18H19N3O3S	103·70	78·34	113·66			
PlateE	A03	MMV676468	TUBERCULOSIS		C10H8NOCl5	96·89	10·46	103·08		YES	
PlateE	B03	MMV676470	TUBERCULOSIS		C18H21NO3	100·33	90·68	114·91			
PlateE	C03	MMV676472	TUBERCULOSIS		C22H17N2O3ClS	92·19	119·54	114·69			
PlateA	H08	MMV676474	TUBERCULOSIS		C21H24N2O2	88·58	60·06	98·99			
PlateA	F07	MMV676476	TUBERCULOSIS		C23H24O5	100·23	72·82	108·09			
PlateA	B11	MMV676477	TUBERCULOSIS		C19H21N5O2S	73·13	4·54	111·26		YES	
PlateC	F09	MMV676478	TUBERCULOSIS		C24H29N5OS	96·53	44·55	111·09			
PlateB	A02	MMV676480	ONCHOCERCIASIS		C26H22N2O5S	104·21	106·26	104·15			
PlateE	G10	MMV676492	LYMPHATIC FILARIASIS		C9H5N3OBrClS2	97·83	83·07	101·07			
PlateA	A09	MMV676501	TUBERCULOSIS		C11H5N3O2Cl2S2	91·78	14·04	103·80		YES	
PlateA	F11	MMV676509	TUBERCULOSIS		C22H17N2O2Cl	89·76	85·87	112·83			
PlateA	H11	MMV676512	TUBERCULOSIS		C18H13N5OS	95·94	6·01	100·17		YES	
PlateA	H10	MMV676520	TUBERCULOSIS		C21H20N2O4	85·79	43·39	100·21			
PlateE	C10	MMV676524	TUBERCULOSIS		C11H10N3Cl	97·02	23·37	113·58		YES	
PlateA	A07	MMV676526	TUBERCULOSIS		C21H17NO3ClF	74·85	97·33	103·45			
PlateB	F09	MMV676528	MALARIA		C20H17N3O3ClFS	102·41	88·72	115·07			
PlateB	D04	MMV676536	SCHISTOSOMIASIS		C18H19N3O2S2	100·73	90·86	119·01			
PlateA	H06	MMV676539	TUBERCULOSIS		C20H16N2O3	95·06	29·71	99·72		YES	
PlateA	G11	MMV676554	TUBERCULOSIS		C19H17N3O3	41·24	29·38	109·41		YES	
PlateA	D08	MMV676555	TUBERCULOSIS		C16H14N3Cl	39·02	71·17	123·00			
PlateA	G07	MMV676558	TUBERCULOSIS		C22H17N3OS	100·23	90·94	110·21			
PlateA	E09	MMV676571	TUBERCULOSIS		C24H20N3O4F	100·47	59·42	115·23			
PlateA	C08	MMV676584	TUBERCULOSIS		C12H8N2OClFS2	90·21	66·80	114·57			
PlateA	G10	MMV676588	TUBERCULOSIS		C15H18N2S	76·50	19·69	101·64		YES	
PlateA	E11	MMV676589	TUBERCULOSIS		C21H23N3O3	88·95	70·07	115·68			
PlateA	G09	MMV676597	TUBERCULOSIS		C18H25N5O2	99·48	97·99	112·65			
PlateE	C05	MMV676599	CRYPTOSPORIDIOSIS		C20H21N5	102·24	88·16	114·91			
PlateB	C03	MMV676600	KINETOPLASTIDS		C26H30N6O3	90·11	91·11	125·11			
PlateB	H02	MMV676602	KINETOPLASTIDS		C25H32N8O	110·68	105·38	95·55			
PlateA	B08	MMV676603	TUBERCULOSIS		C17H16N3O5F3S	100·98	38·43	112·23			
PlateB	B03	MMV676604	KINETOPLASTIDS		C18H21N5O2S	103·73	94·98	118·93			
PlateB	B09	MMV676605	MALARIA		C26H23N5O	109·71	99·45	122·49			
PlateD	G05	MMV676877	MALARIA		C18H18NO2Cl	105·95	90·17	107·29			
PlateD	H05	MMV676881	MALARIA		C14H10N6F2	101·00	98·94	99·43			
PlateC	F05	MMV687138	TUBERCULOSIS		C19H17NO3S	103·58	56·34	101·99			
PlateC	A07	MMV687145	TUBERCULOSIS		C12H17N3O2S	129·84	42·58	107·98			
PlateC	E05	MMV687146	TUBERCULOSIS		C19H26N2O	4·50	77·53	17·24	YES		YES

PlateC	E07	MMV687170	TUBERCULOSIS		C17H13N4O2Cl	89·94	52·71	114·31			
PlateC	H04	MMV687172	TUBERCULOSIS		C23H25N5O	100·70	129·15	98·28			
PlateC	G07	MMV687180	TUBERCULOSIS		C24H25N4O2F3	104·72	74·63	107·33			
PlateC	D07	MMV687188	TUBERCULOSIS		C20H20N2O4	73·54	43·36	110·48			
PlateC	F07	MMV687189	TUBERCULOSIS		C20H20N2O3	98·51	58·08	103·85			
PlateC	A04	MMV687239	TUBERCULOSIS		C19H17N3OF2	97·55	68·07	108·17			
PlateC	C04	MMV687243	TUBERCULOSIS		C15H14NO3Br	105·61	87·08	112·91			
PlateD	C05	MMV687246	MALARIA		C26H25N7O2S	110·38	90·89	118·53			
PlateC	D05	MMV687248	TUBERCULOSIS		C18H13N4OF	109·96	31·83	110·06			
PlateC	C06	MMV687251	TUBERCULOSIS		C8H9N3O4S2	88·32	5·28	116·39		YES	
PlateC	C07	MMV687254	TUBERCULOSIS		C19H19N3O	106·63	105·91	113·25			
PlateC	G06	MMV687273	TUBERCULOSIS		C22H38N2	110·47	31·53	111·35			
PlateC	E06	MMV687696	TUBERCULOSIS		C29H28N4O2ClF3	75·71	44·73	108·81			
PlateC	E04	MMV687699	TUBERCULOSIS		C16H10N3OCl2F3	93·79	53·61	114·27			
PlateE	D10	MMV687700	TUBERCULOSIS		C17H18N6O8S	87·99	84·08	106·99			
PlateC	D04	MMV687703	TUBERCULOSIS		C22H27N5O	3·71	4·55	104·98	YES	YES	
PlateD	A10	MMV687706	KINETOPLASTIDS		C19H20N4O	92·67	99·75	108·13			
PlateE	E08	MMV687729	TUBERCULOSIS		C22H22N2O3F2	30·51	13·14	111·75	YES	YES	
PlateC	C05	MMV687730	TUBERCULOSIS		C22H32N4O2	87·35	71·15	18·76			YES
PlateC	A11	MMV687747	TUBERCULOSIS		C19H17N2O2Br	90·24	63·57	107·94			
PlateC	B05	MMV687749	TUBERCULOSIS		C24H24N6O	96·94	73·32	112·94			
PlateA	D02	MMV687762	KINETOPLASTIDS		C20H20N2O2	101·07	95·85	112·23			
PlateE	C08	MMV687765	TUBERCULOSIS		C25H26N6O	59·04	26·36	115·59		YES	
PlateB	E05	MMV687775	LYMPHATIC FILARIASIS		C19H14NO3BCl2F4	98·15	91·05	122·49			
PlateB	B05	MMV687776	LYMPHATIC FILARIASIS		C28H23N2O4BCl2F4	99·28	90·51	123·97			
PlateE	B02	MMV687794	MALARIA		C15H18N3O2BrS	99·91	90·90	108·63			
PlateE	H03	MMV687796	REFERENCE COMPOUNDS	Amikacin	C22H43N5O13	101·61	9·75	98·96		YES	
PlateE	A05	MMV687798	REFERENCE COMPOUNDS	Levofloxacin (-)-ofloxacin	C18H20N3O4F	1·47	7·23	11·47	YES	YES	YES
PlateB	B06	MMV687800	REFERENCE COMPOUNDS	Clofazimine	C27H22N4Cl2	6·51	6·34	108·77	YES	YES	
PlateB	G04	MMV687801	REFERENCE COMPOUNDS	Ethambutol	C10H24N2O2	31·48	58·86	113·97			
PlateB	H04	MMV687803	REFERENCE COMPOUNDS	Linezolid	C16H20N3O4F	10·81	17·39	24·52	YES	YES	YES
PlateC	F08	MMV687807	TUBERCULOSIS		C15H8NO2ClF6	82·94	4·61	102·82		YES	
PlateD	F11	MMV687812	TUBERCULOSIS		C24H26N8O2F4	40·99	25·97	26·85		YES	YES
PlateE	E10	MMV687813	TUBERCULOSIS		C25H23N3O4F2	34·39	5·59	101·83		YES	
PlateC	A05	MMV688122	TUBERCULOSIS		C16H12N6S	96·70	2·87	110·59		YES	
PlateC	D09	MMV688124	TUBERCULOSIS		C25H37N3O2S	107·26	94·66	116·43			
PlateC	D06	MMV688125	TUBERCULOSIS		C24H33N3O2S	107·06	13·81	113·74		YES	
PlateD	A08	MMV688178	SCHISTOSOMIASIS		C24H17N3O	97·10	103·20	106·82			
PlateC	F03	MMV688179	KINETOPLASTIDS		C18H16N6OCl2	2·34	1·64	17·59	YES	YES	YES

PlateD	B09	MMV688180	KINETOPLASTIDS		C21H24N6O2Cl2S	100·56	93·97	116·42			
PlateC	F06	MMV688262	TUBERCULOSIS	Delamanid	C25H25N4O6F3	112·57	8·60	106·16		YES	
PlateE	A10	MMV688270	SCHISTOSOMIASIS		C22H21N3O2	98·11	83·05	100·64			
PlateD	E10	MMV688271	KINETOPLASTIDS		C18H16N6OCl2	3·73	1·10	108·02	YES	YES	
PlateE	H07	MMV688273	KINETOPLASTIDS		C21H16N4OClF	105·52	97·77	100·32			
PlateD	D06	MMV688274	KINETOPLASTIDS		C27H25N5O3ClF	111·98	85·99	112·67			
PlateD	E09	MMV688279	KINETOPLASTIDS		C23H28N3OCl	113·93	32·52	109·58			
PlateC	C03	MMV688283	KINETOPLASTIDS		C24H23N6O2ClS	112·11	89·42	115·56			
PlateC	H03	MMV688313	SCHISTOSOMIASIS		C23H23N3O2	104·25	101·72	94·75			
PlateC	A08	MMV688327	TUBERCULOSIS	Radezolid	C22H23N6O3F	-1·90	7·39	18·57	YES	YES	YES
PlateE	H02	MMV688330	TOXOPLASMOSIS		C18H19N2O3Cl	102·56	99·58	99·93			
PlateE	D07	MMV688345	TOXOPLASMOSIS		C16H17N5S	86·90	86·05	113·33			
PlateA	H02	MMV688350	DENGUE		C24H20N3O4FS	92·47	105·08	101·64			
PlateE	B11	MMV688352	DENGUE		C29H25N6O2Cl	103·01	89·64	107·06			
PlateA	H03	MMV688360	KINETOPLASTIDS		C25H31N3O3	94·32	100·11	100·24			
PlateC	C09	MMV688361	KINETOPLASTIDS		C21H19N5O	88·91	92·94	116·05			
PlateD	A09	MMV688362	KINETOPLASTIDS		C21H20N4O2	90·24	102·92	108·71			
PlateD	E07	MMV688364	TOXOPLASMOSIS		C25H29N7O2	89·93	82·73	110·53			
PlateC	B11	MMV688371	KINETOPLASTIDS		C22H19N2O2Cl3	106·79	110·91	119·12			
PlateD	G08	MMV688372	KINETOPLASTIDS		C23H20N5OF	111·80	88·82	104·20			
PlateD	D08	MMV688407	KINETOPLASTIDS		C21H21N7O	106·47	94·02	115·66			
PlateC	D02	MMV688410	KINETOPLASTIDS		C21H25N4OCl	104·93	95·32	105·40			
PlateE	C07	MMV688411	TOXOPLASMOSIS		C20H21N7	89·88	90·01	114·40			
PlateE	G11	MMV688415	KINETOPLASTIDS		C26H31N3O3	97·25	88·91	100·72			
PlateA	A04	MMV688416	DENGUE		C23H25N4O2ClS	94·70	99·54	102·51			
PlateC	G05	MMV688417	TOXOPLASMOSIS		C16H15N6Cl	105·71	82·35	103·66			
PlateC	B04	MMV688466	TUBERCULOSIS		C24H24N5O2FS	106·13	93·21	115·10			
PlateD	C08	MMV688467	KINETOPLASTIDS		C22H26N4O2S	111·44	95·50	117·51			
PlateD	H07	MMV688469	TOXOPLASMOSIS		C16H13N5	82·82	90·94	87·32			
PlateA	G02	MMV688470	TOXOPLASMOSIS		C24H28N6O2S	95·67	94·78	106·10			
PlateA	F03	MMV688471	TOXOPLASMOSIS		C21H17N3O2	98·57	87·80	108·78			
PlateA	A03	MMV688472	TOXOPLASMOSIS		C21H16N3O3Cl	92·32	97·99	103·49			
PlateD	F10	MMV688474	KINETOPLASTIDS		C21H21N7O	100·41	94·00	105·80			
PlateC	C02	MMV688508	TUBERCULOSIS		C19H19N2O4F	-0·22	7·31	18·57	YES	YES	YES
PlateC	C08	MMV688509	TOXOPLASMOSIS		C28H26N3O2F	109·53	91·56	118·93			
PlateA	E02	MMV688514	KINETOPLASTIDS		C16H13N3O	100·35	91·65	110·98			
PlateE	F11	MMV688543	DENGUE		C24H46N2O5	97·18	88·96	101·15			
PlateC	B03	MMV688547	KINETOPLASTIDS		C21H21N7O	102·00	95·15	115·71			
PlateA	C03	MMV688548	TOXOPLASMOSIS		C15H17N5	101·12	91·57	114·64			
PlateE	H10	MMV688550	KINETOPLASTIDS		C23H27N5O4	98·96	95·84	105·30			
PlateE	F03	MMV688552	SCHISTOSOMIASIS		C17H14N5OF3S	103·35	81·01	107·45			
PlateA	A08	MMV688553	TUBERCULOSIS		C18H21N3O4	91·35	54·00	104·71			
PlateA	D07	MMV688554	TUBERCULOSIS		C15H19N3O3S	102·20	76·03	115·44			
PlateA	G08	MMV688555	TUBERCULOSIS		C15H18N3O2ClS	92·51	46·77	108·50			
PlateE	G03	MMV688557	TUBERCULOSIS		C12H13NO2S2	100·78	95·72	101·04			
PlateC	G11	MMV688703	TOXOPLASMOSIS		C21H22N3F	103·23	102·27	107·94			

PlateA	G03	MMV688704	TOXOPLASMOSIS		C19H20N4OCl2	98·70	92·85	105·68			
PlateE	G06	MMV688754	KINETOPLASTIDS	Trifloxystrobin	C20H19N2O4F3	103·31	92·90	108·92			
PlateE	F06	MMV688755	TUBERCULOSIS		C14H12N3O5F3	106·79	7·68	111·36		YES	
PlateA	E06	MMV688756	TUBERCULOSIS	Sutezolid	C16H20N3O3FS	2·59	13·36	50·05	YES	YES	
PlateB	A11	MMV688761	SCHISTOSOMIASIS		C20H19N3O6S2	108·09	107·80	112·56			
PlateB	D11	MMV688762	SCHISTOSOMIASIS		C24H23N3	96·21	89·05	121·36			
PlateB	C11	MMV688763	SCHISTOSOMIASIS		C13H10N5OClS2	99·06	21·80	121·66		YES	
PlateE	D02	MMV688766	SCHISTOSOMIASIS		C17H11NO3ClF3	103·92	88·40	111·47			
PlateB	G10	MMV688768	SCHISTOSOMIASIS		C19H23N3S	93·87	86·98	109·23			
PlateE	A04	MMV688771	SCHISTOSOMIASIS		C18H19N3OS	91·68	91·36	98·17			
PlateB	E03	MMV688773	REFERENCE COMPOUNDS	Benznidazole	C12H12N4O3	94·66	82·83	121·51			
PlateB	F03	MMV688774	REFERENCE COMPOUNDS	Posaconazole	C37H42N8O4F2	79·35	100·07	107·18			
PlateE	A06	MMV688775	REFERENCE COMPOUNDS	Rifampicin	C43H58N4O12	1·46	4·71	15·12	YES	YES	YES
PlateA	B05	MMV688776	KINETOPLASTIDS		C17H16N3OCl	100·24	127·06	115·16			
PlateA	D05	MMV688793	KINETOPLASTIDS		C19H19N3O3	101·15	89·74	114·12			
PlateE	B10	MMV688795	KINETOPLASTIDS		C18H23N3O	104·77	77·70	104·80			
PlateA	A06	MMV688796	KINETOPLASTIDS		C19H19N4O2F	93·38	99·33	103·17			
PlateA	E05	MMV688797	KINETOPLASTIDS		C18H22N3O2F	96·45	80·54	113·49			
PlateA	H05	MMV688798	KINETOPLASTIDS		C21H19NO3S	95·21	102·14	102·75			
PlateC	H05	MMV688844	TUBERCULOSIS		C23H25N4O2Cl	4·25	15·44	11·71	YES	YES	YES
PlateC	D10	MMV688845	TUBERCULOSIS		C24H25N3O3S	1·95	6·47	24·10	YES	YES	YES
PlateC	B06	MMV688846	TUBERCULOSIS		C20H21NOClF3	111·53	69·19	20·92			YES
PlateC	A06	MMV688852	TOXOPLASMOSIS		C16H17N5ClF	102·91	107·40	115·14			
PlateB	F06	MMV688853	CRYPTOSPORIDIOSIS		C19H23N5O2	101·88	94·54	110·74			
PlateB	A06	MMV688854	CRYPTOSPORIDIOSIS		C24H28N6O	110·79	110·93	113·89			
PlateA	C04	MMV688888	TUBERCULOSIS		C20H20N5F	37·83	23·32	95·47		YES	
PlateA	B04	MMV688889	TUBERCULOSIS		C19H16N5Cl	89·36	58·90	105·33			
PlateC	G09	MMV688891	TUBERCULOSIS		C18H11NO4BrF3	112·71	101·00	109·08			
PlateC	F11	MMV688921	DENGUE		C23H18N3O5Cl	109·20	109·59	109·65			
PlateA	B06	MMV688934	KINETOPLASTIDS	Tolfenpyrad	C21H22N3O2Cl	104·17	100·81	112·27			
PlateA	F06	MMV688936	TUBERCULOSIS		C18H15N4O2ClS	98·86	82·62	106·00			
PlateE	B04	MMV688938	TUBERCULOSIS		C20H21N2OFS	88·30	119·27	118·09			
PlateE	H04	MMV688939	TUBERCULOSIS		C19H18N4	95·39	48·90	97·60			
PlateD	B11	MMV688941	TUBERCULOSIS		C22H23NO3	103·90	69·85	120·42			
PlateA	D06	MMV688942	KINETOPLASTIDS	Bitertanol	C20H23N3O2	98·60	85·07	111·71			
PlateA	C06	MMV688943	KINETOPLASTIDS	Difenoconazol	C19H17N3O3Cl2	100·24	89·65	111·64			
PlateC	H11	MMV688955	TOXOPLASMOSIS		C15H11N2O5Cl	94·25	103·49	89·90			
PlateA	G05	MMV688958	KINETOPLASTIDS		C18H16N2OS	97·70	91·10	108·61			
PlateE	H05	MMV688978	REFERENCE COMPOUNDS	Auranofin	C20H34AuO9PS	105·17	5·27	106·41		YES	
PlateD	H11	MMV688980	MALARIA		C16H18N3O2FS	92·02	99·57	87·06			
PlateE	H06	MMV688990	REFERENCE COMPOUNDS	Miltefosine	C21H46NO4P	101·76	98·54	102·65			

PlateB	G03	MMV688991	REFERENCE COMPOUNDS	Nitazoxanide	C12H9N3O5S	90·13	82·06	98·31			
PlateB	H11	MMV688994	REFERENCE COMPOUNDS	Streptomycin	C21H39N7O12	80·03	13·66	101·31		YES	
PlateE	B05	MMV689000	REFERENCE COMPOUNDS	Amphotericin B	C47H73NO17	100·63	89·76	114·73			
PlateC	B10	MMV689028	KINETOPLASTIDS		C26H26N4O3S	126·61	120·87	120·75			
PlateC	C10	MMV689029	KINETOPLASTIDS		C26H26N4O4S	93·47	86·92	118·63			
PlateC	B08	MMV689060	KINETOPLASTIDS		C14H18N4Cl2	108·64	104·38	118·59			
PlateC	B09	MMV689061	KINETOPLASTIDS		C24H27N4O2F	126·64	121·78	117·83			
PlateE	E06	MMV689243	KINETOPLASTIDS		C23H20N4F6	109·74	82·64	113·04			
PlateE	C06	MMV689244	KINETOPLASTIDS		C24H21N3OClF3	107·07	90·11	114·62			
PlateB	D06	MMV689255	CRYPTOSPORIDIOSIS	D-Eritadenine	C9H11N5O4	109·48	89·23	123·86			
PlateC	H10	MMV689437	KINETOPLASTIDS		C15H15N7Cl2	106·11	104·17	98·96			
PlateB	G05	MMV689480	REFERENCE COMPOUNDS	Buparvaquone	C21H26O3	95·51	98·52	111·73			
PlateC	E09	MMV689709	KINETOPLASTIDS		C19H21N5O2	91·66	97·32	114·42			
PlateA	A05	MMV689758	REFERENCE COMPOUNDS	Bedaquiline	C32H31N2O2Br	1·81	5·64	19·76	YES	YES	YES
PlateB	C02	MMV690027	KINETOPLASTIDS		C33H40N6O4	103·87	100·69	111·20			
PlateA	C05	MMV690028	KINETOPLASTIDS		C30H38N6O4	92·29	95·80	120·36			
PlateC	E08	MMV690102	KINETOPLASTIDS		C22H23N7O2	110·89	106·31	112·53			
PlateC	D08	MMV690103	KINETOPLASTIDS		C19H23N7	110·26	104·75	117·19			

Table S2: Physicochemical properties of four hit compounds with MICs determined against *M.chimaera*.

Data taken from [a] https://www.mmv.org using MMV identifiers; and [b] https://www.ebi.ac.uk/chembl using corresponding SMILES entries.

Compound ID[a]	Disease Set[a]	Molecular Weight[a]	Molecular Formula[a]	cLogP[a]	Chemical Class[a]	SMILES[a]	chEMBL ID[b]	#Rule of Five Violations (Lipinski)[b]
MMV022478	Malaria	545·93	C23H21N6OCl	2·55	Pyrazolopyrimidine	Clc1cccc(c1)c2cnn3ccc(nc23)C(=O)Nc4ccc(cc4)N5CCNCC5	534797	0
MMV675968	Cryptosporidiosis	359·81	C17H18N5O2Cl	2·31	Diaminoquinazoline	COc1ccc(OC)c(NCc2ccc3nc(N)nc(N)c3c2Cl)c1	88430	0
MMV688179	Kinetoplastid	476·19	C18H16N6OCl2	2·87	Bisarylguanidinium	NC(=N)Nc1ccc(c(Cl)c1)c2oc(cc2)c3ccc(NC(=N)N)cc3Cl	1788168	1
MMV688271	Kinetoplastid	476·19	C18H16N6OCl2	2·65	Bisarylguanidinium	NC(=N)Nc1ccc(cc1Cl)c2oc(cc2)c3ccc(N=C(N)N)c(Cl)c3	413331	1

Table S3: Cytotoxicity and drug metabolism pharmacokinetics data of four hit compounds with MICs determined against *M.chimaera.*

Detailed from https://www.mmv.org using MMV identifiers. HL60: human promyelocytic leukaemia cell line; MRC5: human fibroblast cell line derived from foetal lung tissue; HepG2: human hepatocellular carcinoma cell line; CC20, CC50: cytotoxic concentration required to reduce cell viability by 20% and 50% respectively; IC50: concentration required to cause inhibition by 50%; hERG: human Ether-à-go-go-Related Gene; CYP: cytochrome P450; PAMPA: parallel artificial membrane permeability assay; GSH: glutathione.

Compound ID	HL60 CC50 (µM)	MRC5 CC50 (µM)	HepG2 CC20 (µM)	HepG2 CC20 (µM)	hERG Thallium flux IC50 (µM)	CYP1A2 IC50 (µM)	CYP2C9 IC50 (µM)	CYP2D6 IC50 (µM)	CYP3A4 IC50 (µM)	PAMPA (10e-6 cm/sec)	Mouse plasma stability (%)	Glutathione reactivity (classification)
MMV022478	Not Tested	Not Tested	3·4	0·764	30	7·65	>20	>20	7·88	Not Tested	Not Tested	No GSH adduct
MMV675968	>25	Not Tested	3·4	3·44	11	>20	9·54	>20	10·4	0·276	97	Very low
MMV688179	Not Tested	11·6	6·8	6·75	>30	2·93	>20	Not Tested	2·15	Not Tested	Not Tested	Medium
MMV688271	Not Tested	15·4	13·5	13·5	>30	1·29	10·2	Not Tested	2·05	Not Tested	Not Tested	No GSH adduct

References

1. Medicines for Malaria Venture. About the Pathogen Box. https://www.mmv.org/mmv-open/pathogen-box/about-pathogen-box (accessed August 04 2021).

2. Woods GL, Brown-Elliott BA, Conville PS, et al. Susceptibility Testing of Mycobacteria, Nocardiae, and Other Aerobic Actinomycetes. In: CLSI Standards: Guidelines for Health Care Excellence, 2nd ed. Philadelphia: Clinical and Laboratory Standards Institute, 2011: 19-26.

3. Low JL, Wu ML, Aziz DB, Laleu B, Dick T. Screening of TB Actives for Activity against Nontuberculous Mycobacteria Delivers High Hit Rates. Front Microbiol 2017; 8: 1539.
